# *Streptococcus pneumoniae* in Biofilms Are Unable to Cause Invasive Disease Due to Altered Virulence Determinant Production

**DOI:** 10.1371/journal.pone.0028738

**Published:** 2011-12-08

**Authors:** Carlos J. Sanchez, Nikhil Kumar, Anel Lizcano, Pooja Shivshankar, Julie C. Dunning Hotopp, James H. Jorgensen, Hervé Tettelin, Carlos J. Orihuela

**Affiliations:** 1 Department of Microbiology and Immunology, The University of Texas Health Science Center at San Antonio, San Antonio, Texas, United States of America; 2 Department of Microbiology and Immunology, Institute for Genome Sciences, University of Maryland School of Medicine, Baltimore, Maryland, United States of America; 3 Department of Pathology, The University of Texas Health Science Center at San Antonio, San Antonio, Texas, United States of America; University of Kansas Medical Center, United States of America

## Abstract

It is unclear whether *Streptococcus pneumoniae* in biofilms are virulent and contribute to development of invasive pneumococcal disease (IPD). Using electron microscopy we confirmed the development of mature pneumococcal biofilms in a continuous-flow-through line model and determined that biofilm formation occurred in discrete stages with mature biofilms composed primarily of dead pneumococci. Challenge of mice with equal colony forming units of biofilm and planktonic pneumococci determined that biofilm bacteria were highly attenuated for invasive disease but not nasopharyngeal colonization. Biofilm pneumococci of numerous serotypes were hyper-adhesive and bound to A549 type II pneumocytes and Detroit 562 pharyngeal epithelial cells at levels 2 to 11-fold greater than planktonic counterparts. Using genomic microarrays we examined the pneumococcal transcriptome and determined that during biofilm formation *S. pneumoniae* down-regulated genes involved in protein synthesis, energy production, metabolism, capsular polysaccharide (CPS) production, and virulence. We confirmed these changes by measuring CPS by ELISA and immunoblotting for the toxin pneumolysin and the bacterial adhesins phosphorylcholine (ChoP), choline-binding protein A (CbpA), and Pneumococcal serine-rich repeat protein (PsrP). We conclude that biofilm pneumococci were avirulent due to reduced CPS and pneumolysin production along with increased ChoP, which is known to bind C-reactive protein and is opsonizing. Likewise, biofilm pneumococci were hyper-adhesive due to selection for the transparent phase variant, reduced CPS, and enhanced production of PsrP, CbpA, and ChoP. These studies suggest that biofilms do not directly contribute to development of IPD and may instead confer a quiescent mode of growth during colonization.

## Introduction

*Streptococcus pneumoniae* (the pneumococcus) is a leading cause of otitis media, community-acquired pneumonia, sepsis and meningitis. *S. pneumoniae* typically colonizes the human nasopharynx asymptomatically with invasive pneumococcal disease (IPD) occurring as a result of dissemination to, and bacterial replication at, normally sterile sites including the middle ear, lungs, and bloodstream. IPD is opportunistic in nature and primarily occurs in infants, the elderly, and those with underlying medical conditions [1], [2], [3], [4]. Worldwide the pneumococcus is responsible for more than 14.5 million episodes of IPD annually and up to 11% of all deaths in children [5], [6]. Notably, in individuals >65 years of age the case-fatality rate for IPD can be as high as 30% [7]. Thus pneumococcal infections are a major medical problem for both children and the elderly.

*S. pneumoniae* biofilm formation has been shown to occur in humans during nasopharyngeal colonization and recurrent otitis media. Pneumococcal biofilms have been detected in human sinus mucosa biopsies, resected adenoids from individuals with tonsillitis, and biofilms have been observed within tympanostomy tubes collected from children with chronic otitis media [8], [9]. Fulfilling Koch's postulates, biofilms and biofilm-like pneumococcal aggregates have been observed in the middle ears of experimentally infected chinchillas as well as bronchial and nasal lavage fluids taken from the nasopharynx of infected mice, respectively [10], [11]. Thus biofilm formation is a naturally occurring, if not yet fully understood, biological mechanism for *S. pneumoniae*.

During the past 10 years considerable effort has gone towards dissecting the molecular mechanisms underlying biofilm development *in vitro* and its recalcitrance to antimicrobial therapy *in vivo* [12], [13], [14], [15], [16]. Importantly, and despite these considerable findings, whether biofilm formation contributes towards the development of IPD remains unclear. For example, studies by Munoz-Elisa *et al.*, Parker *et al.*, and Trappetti *et al.*, indicate that genes required for robust biofilm formation *in vitro* are important for nasopharyngeal colonization and in some instances progression towards lung disease [15], [17], [18], [19]. In contrast, studies by Tapianen *et al.*, Camilli *et al.*, and ourselves, have found no correlation between the ability of isolates to form robust biofilms *in vitro* and virulence potential in humans and mice [12], [20], [21]. Thus, experiments directly testing the virulence potential of pneumococcal biofilms are needed to confirm or disprove their role during IPD.

In this study, we show that biofilm pneumococci are capable of colonizing the nasopharynx yet unable to cause invasive disease. We show this to be in part the result of altered production of capsular polysaccharide (CPS) [22], pneumolysin [23], cell wall phosphorylcholine (ChoP) [24], Choline binding protein A (CbpA) [25], and Pneumococcal serine-rich repeat protein (PsrP) [26]. Our findings suggest a limited role for biofilms during IPD and provide information on how biofilm pneumococci might modulate their interactions with the host during nasopharyngeal colonization to support long-term quiescent colonization. Importantly, due to altered virulence determinant production by biofilm pneumococci, our findings have important implications towards the selection of protein antigens for any next-generation vaccine against *S. pneumoniae*.

## Methods

### Bacterial strains and growth conditions

*Streptococcus pneumoniae* serotype 4, strain TIGR4, T4R its unencapsulated derivative, T4 *ΔpsrP* a *psrP* deficient mutant, R6 an un-encapsulated serotype 2 laboratory strain, A66.1 a serotype 3 isolate, and all the clinical isolates used in this study have been previously described [22], [27], [28]. Bacterial strains were grown on tryptic soy blood agar plates (Remel, USA) at 37°C in 5% CO_2_. For planktonic growth, Todd Hewitt Broth (THB) was inoculated with overnight plate cultures and grown to mid-logarithmic phase (OD_620_ = 0.5; ∼1.0×10^8^ CFU/ml) using normal culture conditions. Mature *S. pneumoniae* biofilms were grown under once-through flow conditions using a once-through biofilm line reactor, as previously described [11]. Briefly, planktonic seed cultures were used to inoculate 1 meter long silicone tubing (0.89 mm internal diameter, Cole Parmer Inc.). Bacteria in the line were allowed to attach for 2 h after which the flow rate of media was adjusted to 0.035 ml/min. Bacterial biofilms were grown for up to 2 days at 37°C in 5% CO_2_. Biofilm-derived bacteria were harvested from the line by pinching the tube along its entire length, thereby removing the bacterial cells. Frozen stocks of both biofilm and planktonic derived bacteria were made in THB containing 12% glycerol (vol/vol) and stored at −80°C. For animal experiments with biofilm-derived planktonic pneumococci, glass test tubes containing THB were inoculated with biofilm pneumococci at 10^5^ colony forming units (CFU)/ml. Similar to the planktonic cultures, at mid-logarithmic growth phase frozen stocks were created and stored. In all instances viable bacterial CFU counts were determined by thawing aliquots, and plating serial dilutions.

### Scanning and transmission electron microscopy images of mature biofilms

Following growth of biofilms within the biofilm reactor lines, the line containing the biofilms were cut in half to expose the lumen, fixed for 2 h with 2.5% glutaraldehyde in PBS, and then rinsed twice for 3 min in 0.1 M phosphate buffer (pH 7.4). Samples were submerged in 1% osmium tetroxide diluted in Zetterquist's Buffer for 30 min then washed with the same buffer for 2 min. This was followed by stepwise dehydration with ethanol (i.e. 70%, 95%, and 100%); the first two steps for 15 min, the last for 30 min. Samples were treated with hexamethyldisilizane for 5 min prior to drying in a desiccator overnight. The next day samples were sputter coated with gold palladium and viewed with a JEOL-6610 scanning electron microscope [11]. For transmission electron microscopy, biofilm derived bacteria grown under once through conditions as above were harvested at the indicated time points and fixed with a fixation solution containing 4% formaldehyde and 1% glutaraldehyde for 1 h. Following several washes the samples were then dehydrated with a graded series of acetone (10, 30, 50, 70, 90, and 100%) on ice for 15 min for each step and embedded within acrylic resin. Ultrathin sections of samples were cut with a diamond knife, and placed onto Formvar-coated copper grids (300 mesh). Counterstaining of the sections was performed with 4% aqueous uranyl acetate for 5 min. After air-drying, samples were examined with a JEOL 100CX transmission electron microscope.

### Antimicrobial susceptibility of biofilm and planktonic bacteria

The effect of various antimicrobials was tested on biofilm and planktonic bacteria in 96-well flat bottom plates using a modified version of the standard microdilution assay including: erythromycin (0.015–32 µg/ml), clindamycin (0.015–32 µg/ml), penicillin (0.03–8 µg/ml), cefazolin (0.03–8 µg/ml), and vancomycin (0.06–4 µg/ml) [29]. Briefly, planktonic or biofilm derived bacteria were suspended in 10 µl of 0.85% saline and directly inoculated into 96 well plates containing a pre-diluted antibiotic in 100 µl of Cation-adjusted Mueller-Hinton Broth enriched with 3% lysed horse blood such that the final titer was 10^5^ CFU/ml. The plates were then incubated at 37°C for 5 h. Following incubation the content of each well was diluted and plated onto blood agar plates for colony counting. Bacterial susceptibility was determined by measuring the concentration for which <10^3^ CFU/ml were thereafter viable. All experiments were performed in triplicate and the results are expressed as the average values.

### Virulence studies in mice

All animal experimentation were conducted following the National Institutes for Health guidelines for housing and care of laboratory animals. Animal experiments were reviewed and approved by the Institutional Animal Care and Use Committee at The University of Texas Health Science Center at San Antonio; protocol number 09022-34. Female BALB/cJ mice (The Jackson Laboratories) of 5 to 6 week of age were anesthetized with 2.5% isoflurane and infected with either planktonic, biofilm, or biofilm-derived planktonic pneumococci suspended in PBS. For intranasal challenge (n = 10/cohort), each mouse was infected drop wise into the left nare with 10^6^ CFU in 25 µl of PBS. On days 1, 3, and 5 bacterial titers in the nasopharynx and blood were determined by nasopharyngeal lavage with 10 µl saline or collection of blood from the tail vein and plating of serial dilutions, respectively. For intratracheal challenge (n = 6–9/cohort) 10^5^ CFU in 100 µl PBS was instilled into the lungs by forced aspiration; aspiration was induced by gently pulling the tongue of anesthetized mice outward, placing the bacterial suspension in the throat, and covering the nostrils. Mice were sacrificed 24 h post-challenge and bacterial titers in the lungs and blood determined. Bacterial titer in the lungs was determined by plating serial dilutions of lung homogenates and normalized per gram of total tissue. Finally, for intraperitoneal challenge (n = 6–7/cohort), mice were injected with 10^4^ CFU in 100 µl PBS using a 27-gauge needle. Bacterial titers in the blood were determined at 24 h post challenge by plating of serial dilutions of blood collected from the tail vein.

### Bacterial adhesion assays

A549 and Detroit 562 cells (ATCC, Manassas, VA) were maintained in F-12 media supplemented with 10% fetal bovine serum and in Minimal Essential Medium supplemented with 10% fetal bovine serum and 0.2% lactoalbumin hydrolase respectively. All cell lines were maintained at 37°C in 5% CO_2_. Adhesion assays were performed as previously described [30]. A549 cells and Detroit 562 cells were grown to 95% confluence in COSTAR 24-well polystyrene plates (∼10^6^ cells/well). Cells were washed with sterile phosphate buffered saline (pH 7.4) and exposed to F12 media without serum containing 10^7^ CFU/ml of either biofilm or planktonic derived bacteria diluted from the frozen stocks. Cells were incubated for 1 h at 37°C in 5% CO_2_. Following incubation, non-adherent bacteria were removed by gently washing the cells three times with PBS and the number of adherent bacteria was determined by lysis of the cell monolayer with 0.1% Triton X-100 in PBS and plating the lysates on blood agar plates. Each experiment contained 3 biological replicates per condition and was repeated ≥3 times. Adhesion is expressed as a percentage compared to the planktonic counterpart.

### Isolation of pneumococcal RNA

*S. pneumoniae* grown under biofilm conditions for 4, 12, 24, and 48 h were collected, immediately suspended in RNAprotect (Qiagen) and stored at −20°C. Isolation of bacterial RNA was performed using an RNeasy Mini kit (Qiagen) following the manufacturer instructions with exception to bacteria lysis. Briefly, bacterial cells were suspended in 350 µl buffer RLT and transferred to 2 ml cryogenic safe-lock tubes containing approximately 25 mg of 0.1 mm zirconia/silicon acid washed beads (BioSpec). Cells were lysed by mechanical disruption using a Bead-Beater (BioSpec) for 5 min at maximum speed. Following lysis, beads were removed by passage through a QiaShredder column (Qiagen) and the supernatant was transferred into a 2 ml tube containing an equal volume of ethanol (70%). The lysate was transferred directly onto an RNeasy Mini spin column and bacterial RNA was purified as per the kit's directions. Control RNA samples were generated from the seed cultures used to inoculate the biofilm lines (OD_620_ = 0.5) at 37°C in 5% CO_2_. RNA quality and quantity was determined by (i) measurement of absorbance at 260/280 nm, (ii) visualization of RNA samples using a 1% formaldehyde gel, and (iii) analyzing RNA profiles generated on the Agilent 2100 Bioanalyzer (Agilent Technologies, Germany) using Prokaryote Total RNA Nano chips.

### Microarray analysis of pneumococcal gene transcription and analysis of hybridization data

The *S. pneumoniae* microarrays used in this study consisted of 3482 70-mer oligonucleotide probes from the genome of 3 pneumococcal strains (TIGR4, R6 and G54) as well as 10 amplicons and 500 oligonucleotides (70-mers) from *Arabidopsis thaliana* which served as negative controls. Probes were printed 3× on aminosilane-coated slides (SCHOTT Nexterion). The microarrays (version 8) were kindly provided by the Pathogen Functional Genomics Resource Center (http://pfgrc.jcvi.org/index.php/microarray/array_description/streptococcus_pneumoniae/version8.html) and experiments were performed as previously described [31].

Aliquots of 2 µg of the total RNAs were reverse transcribed into single-stranded cDNA using 200 U Superscript II reverse transcriptase (Invitrogen), 6 µg random hexamers (Invitrogen), 1× first strand buffer (Invitrogen), 10 mM dithiothreitol (DTT), 0.5 mM dATP, 0.5 mM dCTP, 0.5 mM dGTP, 0.3 mM dTTP and 0.2 mM of aminoallyl-modified nucleotide (Invitrogen). The mixture was incubated overnight at 42°C and the reaction stopped by addition of 10 µl 0.5 M EDTA and 10 µl 1 M NaOH. Amine-modified cDNA was purified using QIAquick PCR purification kit (QIAGEN) followed by chemical labeling with Cy3- or Cy5-NHS-ester fluorescent dyes (GE Healthcare, Piscataway, NJ) in a final step.

Slides were prehybridized in a 50 ml solution of 5× SSC, 0.1% SDS and 1% BSA for at least 1 h at 42°C, washed 10× in water and once in isopropanol, then dried by brief centrifugation. Labeled probes were re-suspended in hybridization buffer (50% formamide, 5× SSC, 0.1% SDS, 1 µL 0.1 M DTT, 0.6 µg/µL salmon sperm DNA) and hybridized to the microarray slides in a 42°C water bath for 16–20 h. Slides were washed twice in a low stringency buffer (2× SSC, 0.1% SDS) at 55°C for 5 min, twice in a medium stringency buffer (0.1× SSC, 0.1% SDS) at room temperature for 5 min, twice in a high stringency buffer (0.1× SSC) at room temperature for 5 min, and finally in water for 2 min, and then dried by brief centrifugation.

Synthesized cDNA from each RNA sample from 3 (4 and 12 h time points) or 2 (24 and 48 h time points) independent preparations was hybridized on separate microarray slides (biological replicates), and independently synthesized cDNA from each of these RNA samples was hybridized in a repeat dye-swap experiment (technical replicates, except for the 48 h time point where RNA quantities were limiting) to test technical reproducibility.

Hybridized slides were scanned using a GenePix 4000B dual-color laser scanner (Axon Instruments, CA, USA). Signal intensities, generated using TIGR Spotfinder program version 2.2.3, http://www.tm4.org/spotfinder.html) [32], were imported into TIGR MIDAS software (v2.19) for filtering and normalization. Spots with Cy3 or Cy5 fluorescence intensities <10,000 were discarded. Intensities were normalized using iterative log-mean centering. Data from replicate experiments (only where *n*≥7, except for the 48 h time point where *n*≥4) were averaged using in-house developed Perl scripts. Data points that did not meet these requirements are labelled “NA” in Tables S1, S2 and S3. Oligonucleotides with NA for all conditions tested were omitted from the tables. The Gene Expression Omnibus (GEO, http://www.ncbi.nlm.nih.gov/geo/) series accession number for the microarray data of this study is GSE26976. The significance of ratios of query (e.g. 4 h time point) over the reference (culture prior to inoculation of the biofilm reactor) was assessed using a one-class Student *t*-test with means of log_2_ of ratios tested against 0, p-values based on *t*-distribution with an overall threshold of 0.01 and Bonferroni correction.

### Confirmation of microarray expression levels with qRT-PCR

Real-time quantitative reverse transcription-PCR (qRT-PCR) was performed in a two-step reaction consisting of reverse transcription and real-time PCR. Reverse transcription was carried out using the QuantiTect Reverse Transcription Kit (Qiagen) in accordance with the manufacturer's instructions. Briefly, 1 µg of total RNA was incubated in gDNA Wipeout Buffer (7×) and RNase-free water and incubated at 42°C for 2 min to remove contaminating genomic DNA. The cDNA was synthesized from the RNA using Quantiscript reverse transcriptase, Quantiscript RT buffer and a primer mix consisting of long random primers and oligo-dT. The reaction was incubated at 42°C for 15 min and then at 95°C for 3 min to inactivate Quantiscript reverse transcriptase. Quantitative real-time PCR was performed as previously described [31]. Dilutions of the cDNA (0.2 µl of stock cDNA per 20 µl reaction) were used as template in a reaction containing 2× QuantiTect SYBR Green mix (Qiagen), RNase-free water and 20 gene-specific primers. The gene-specific primers were designed using Primer3 and synthesized by Eurofins MWG Operon (Alabama, USA). The qRT-PCR was conducted using an ABI 7900HT machine (Applied Biosystems). The reactions were denatured at 95°C for 15 min followed by amplification with 45 cycles of 94°C for 15 s, 55°C for 30 s and 72°C for 30 s. Reactions were followed by a melt curve analysis that starts at 55°C, with a dissociation step at 95°C for 1 min plus 0.5°C/cycle for 80 cycles.

The qRT-PCR data was analyzed using a comparative cycle threshold (ΔCt) method [33]. The ΔCt was normalized to genes that did not exhibit any significant change in expression as identified by the microarray experiments. Each sample from each biological replicate was analysed twice (technical replicates).

### Quantification of CPS and teichoic acid

Levels of CPS and teichoic acid containing ChoP were determined using ELISA as previously described with minor modifications [34]. Stocks of pneumococci grown under biofilm and planktonic conditions were suspended in 1 ml of PBS and sonicated for three 10 s intervals on ice prior to storage at −20°C. Total cellular protein was determined by bicinchoninic acid (BCA) assay (Sigma) as per the manufactures instructions. Bacterial sonicates at the designated protein concentrations were diluted in a sodium bicarbonate/carbonate buffer (100 mM, pH 9.6) and fixed overnight to 96-well polystyrene plates (Nunc Maxisorp, Apogent, USA) at 4°C. The plates were blocked with 1% BSA in PBS (blocking buffer) for 2 h at room temperature then washed 3 times with PBS. For detection of serotype 4 CPS, type specific rabbit antiserum against serotype 4 capsule (Statens Serum Institut, Denmark) was added at 1∶20,000 in blocking buffer, and incubated at room temperature for 1 h. Binding of type-specific antiserum, was detected by washing, incubation with an HRP- conjugated goat anti rabbit antibody at 1∶10,000 in blocking buffer, washing, and development by standard methods using tetramethylbenzidine and hydrogen peroxide as the substrate reagent. Plates were read using a plate reader at 450 nm. Relative amounts of ChoP were detected as above with some modifications. For quantification of teichoic acid mouse monoclonal IgA antibody (Clone ID TEPC-15, Cat# M1421, Sigma) specific ChoP was used at 1∶5,000 and binding of primary antibody was detected using an alkaline phosphatase conjugated goat anti-mouse IgA (1∶10,000) (Cat# 1040-04, Southern Biotech, USA). Levels of teichoic acid were also confirmed by Western blot analysis. Equal amounts of whole bacterial cell sonicates (15 µg) as measured by BCA assay were separated on a 15% SDS-PAGE gel and electrophoretically transferred to nitrocellulose membranes using standard methods. Membranes were blocked with 4% bovine serum albumin (BSA) in PBS for 2 h at room temperature then incubated overnight at 4°C with mouse monoclonal antiserum (TEPC-15) specific for ChoP at a dilution of 1∶10,000. Alkaline phosphatase conjugated goat anti-mouse IgA at 1∶5,000 was used to detect the primary antibody and NBT/BCIP Ready-to Use Tablets (Roche) were used as the alkaline phosphatase substrate for development. Equal loading of sonicates was confirmed by Coomassie brilliant blue staining of parallel loaded gels, and the staining of membranes with Ponceau stain (Sigma) following immunoblot. For ELISA experimental values are expressed as the average of a minimum of three independent experiments performed in triplicate. For consistency immunoblots were performed three times.

### Immunoblot assays

Whole cell bacterial lysates were prepared by sonication of the samples on ice for three 10 s intervals. Total cellular protein was determined by bicinchoninic acid (BCA) assay (Sigma) as per the manufacturer's instructions. Whole cells lysates (25 µg) were separated by 12% SDS-PAGE and electrophoretically transferred to nitrocellulose membranes. For PsrP, samples were directly blotted onto nitrocellulose membranes. Membranes were blocked with PBS containing 4% bovine serum albumin (BSA) and 0.1% Tween-20 for 1 h and incubated overnight at 4°C with rabbit sera to PsrP or CbpA (a gracious gift from Elaine Tuomanen, Memphis TN), or mouse monoclonal sera to or pneumolysin (NCL-SPN; Novocastra Laboratories) or ChoP. A HRP-conjugated secondary antibody was used for detection of the proteins by chemiluminesence. Equal loading of sonicates was confirmed by Coomassie brilliant blue staining of parallel loaded gels, and the staining of membranes with Ponceau stain (Sigma) following immunoblot.

### Determination of phase variant phenotype

Phase variation in pneumococci was assessed as previously described [24]. Pneumococci were streaked onto tryptic soy broth (TSB) plates supplemented with 1% agar onto which 100 µl of catalase (6300 U) (Sigma) was added. Plates were incubated at 37°C in 10% CO_2_ for 16 h. Following incubation colony morphologies (i.e. phase variation) were assessed under magnification and oblique transmitted illumination by differential interference contrast (DIC) microscopy using a Zeiss Axiovision Imager Z1. Frequency of phase variants was determined by counting and determining the phenotype of ≥100 random colonies from at lest 6 plates with either seed culture or biofilm derived TIGR4. Colony images were captured using a Leica S6D light microscope and digital camera.

### Statistical analysis

For pair-wise comparisons of groups statistical analyses were performed using a two-tailed Student's *t*-test. For multivariate analyses a 1-Way ANOVA followed by a post-priori test using Sigma Stat software was used. For survival studies a Kaplan Meier Log-Rank test was used. Values were determined to be statistically significant if the *P*<0.05.

## Results

### Verification of mature pneumococcal biofilm formation

**Figure 1:**
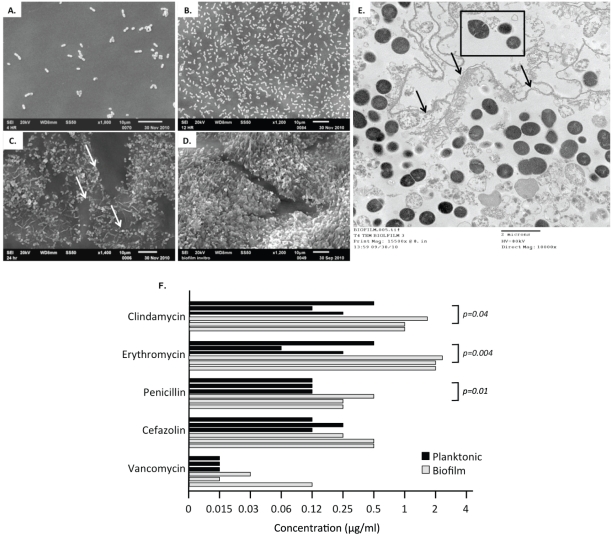
Visual characterization of *S. pneumoniae* mature biofilm development *in vitro*. Scanning electron microscipy images representative (n = 6) of mature *S. pneumoniae* (TIGR4) biofilms developed *in vitro* under once through conditions. Images of biofilm growth at 4 h (A), 12 h (B), 24 h (C) and 48 h (D) are depicted. Transmission electron microscopy image of a cross section of a mature pneumococcal biofilm grown for 48 h is shown (E). Note the demarcation of the outer matrix edge (black arrows), the presence of viable and dead pneumococci enveloped within the matrix, and the presence of surface exposed diplococci available for dispersal (within black box). (F) Tolerance to the killing effect of the designated antimicrobials was determined by measuring the concentration for which >10^3^ CFU were viable after 5 h's of incubation with the antibiotic. Wells were inoculated with 10^5^ CFU/ml. Results from three independent experiments are shown. Statistical analysis was performed using a two-tailed Student's *t-*test.

We first utilized electron microscopy to verify that pneumococci growing within the bioreactor lines were indeed in a mature biofilm state. SEM of TIGR4 revealed that biofilm formation occurred in a series of steps including initial attachment, formation of a “bacteria lawn”, then the striking incremental formation of a large biofilm matrix (Figure 1A–D). By 24 h, pneumococci had begun to form an extracellular polymeric matrix (EPM). At 48 h, EPM was the main component of the biofilm, encompassing most but not all pneumococci (Figure 1E). TEM analysis determined that the mature biofilm was predominantly acellular and primarily composed of dead pneumococci (Figure S1A). At 24 h only 67% of biofilm pneumococci were presumably viable (having an intact membrane and discernible electron dense body) and by 48 h only 27% were intact (Figure S1B). Thus, we determined that a major component of the mature biofilm and by extension the EPM was remnants of dead bacteria.

A key characteristic of biofilms is their recalcitrance to antimicrobials [35], [36]. We tested the resistance of pneumococci isolated from the biofilm reactor using a modified version of the broth antimicrobial microdilution assay (Figure 1F) [29]. Typically antibiotic resistance assays are performed over a 16–24 hour period, this was not done as the biofilm phenotype would be lost over this extended time-period and a shortened testing period has been accepted as a viable method for testing the biofilm phenotype [37]. In contrast to TIGR4 grown in a planktonic state, biofilm derived bacteria were highly tolerant to a 5 h exposure to erythromycin, clindamycin, and to a modest level of penicillin. Biofilm and planktonic bacteria were equally susceptible to cefazolin and vancomycin. Notably, the observed tolerence to protein translation inhibitors was consistent with other published biofilm studies [16], [29]. Thus these experiments served as verification that our biofilm model was valid and that we would be examining mature biofilm bacteria in our subsequent experiments.

### Biofilm-derived bacteria are avirulent

**Figure 2:**
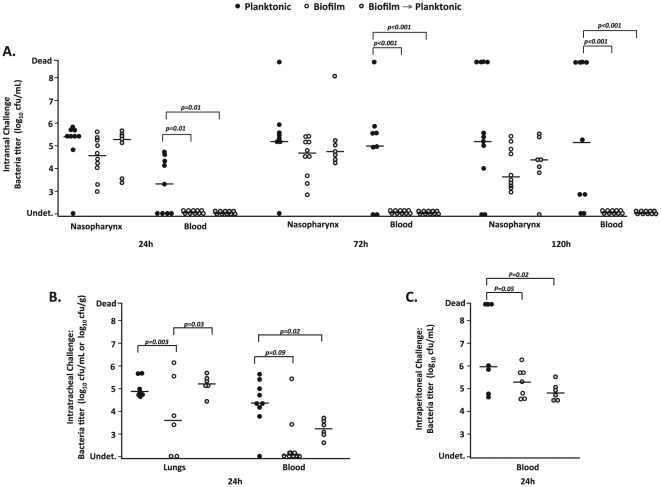
Biofilm-derived pneumococci are unable to cause invasive disease. Bacterial titers were measured for individual 5-week old female BALB/cJ mice infected with either planktonic (dark circles), biofilm-derived (white circles), or biofilm-derived planktonic (grey circles) *S. pneumoniae* TIGR4 via intranasal (10^6^ CFU; n = 10) (A), intratracheal (10^5^ CFU; n = 6–8) (B), or intraperitoneal (10^4^ CFU n = 6–8) (C) routes. For intranasal challenge, nasal lavages and blood were collected at 24, 72, and 120 h post-infection. For intratracheal and intraperitoneal challenge, samples were collected at 24 h post-infection. Horizontal bars represent the median value. Statistical analysis was performed using a two-tailed Student's *t-*test.

Having confirmed our ability to grow mature biofilms, we tested whether biofilm pneumococci were virulent when compared to their planktonic counterparts. Following intranasal challenge of mice with equivalent CFU of TIGR4, no difference between the biofilm and planktonic cultures could be discerned in nasal lavage counts (Figure 2A), or in their ability to form the previously described biofilm-like aggregates within the nasopharynx (n = 6/cohort; data not shown) [11]. Importantly, only those mice that received planktonic pneumococci proceeded to develop bacteremia and died. At 24, 72 and 120 h 4, 7, and 7 of the 9 mice infected intranasal with planktonic bacteria (or 44%, 77%, and 77% respectively) had either positive blood cultures or had succumbed to infection. In contrast, none of the biofilm-infected animals had detectable bacteria in the blood at any time tested. This difference in virulence potential was verified by intratracheal challenge of mice (Figure 2B). All mice receiving planktonic bacteria developed pneumonia and bacteremia, whereas the majority of the mice infected with biofilm derived bacteria had low levels of bacteria in their lungs and no detectable levels of bacteria in the blood. The biofilm-infected mice all successfully cleared the infection. Finally, 24 h after intraperitoneal challenge, 43% of the mice challenged with planktonic bacteria had died, whereas all of the biofilm bacteria remained alive (Figure 2C). Thus, despite challenge with equal bacterial titers as determined by CFU, mice infected with biofilm-derived pneumococci cleared infection, indicating that the biofilm phenotype was attenuated for invasive disease but not for colonization.

### Biofilm pneumococci are hyper-adhesive

**Figure 3:**
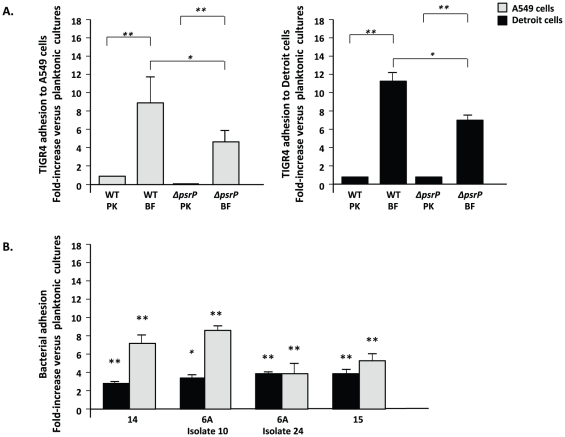
Biofilm-derived pneumococci have an enhanced ability to adhere to host cells. (A) Comparison of the adhesive properties of planktonic bacteria and biofilm-derived *S. pneumoniae* TIGR4 and T4Δ*psrP* to A549 cells and Detroit 562 cells *in vitro*. (B) Bacterial adhesion assays using a panel of invasive clinical isolates of *S. pneumoniae* unrelated to TIGR4. Values are expressed as fold-increase adhesion relative to the planktonic counterparts. All experiments were performed in triplicate and repeated independently at least three times. Statistical analysis was performed using a two-tailed Student's *t-*test. Single asterisks denotes *P*<0.001, double asterisks denotes *P<0.00001*.

We tested whether the decreased virulence of biofilm bacteria was due to an inability to bind epithelial cells. Biofilm-derived bacteria had a 9-fold and 12-fold greater ability to adhere to A549 and Detroit 562 cells versus their planktonic counterparts, respectively (Figure 3A). Using clinical isolates of 3 additional serotypes (i.e. 6A, 14 and 15), we confirmed that the hyper-adhesive biofilm state occurred in both a serotype and strain-independent manner to both cell lines (Figure 3B). Thus, an inability to attach to host cells was not responsible for the attenuated virulence of biofilm pneumococci.

### Temporal biofilm-related changes in gene expression

To determine why biofilm pneumococci were avirulent we compared pneumococcal gene expression after 4, 12, 24 and 48 h of biofilm growth versus that of planktonic exponential phase cultures using microarrays. RNA isolated from our exponential phase TIGR4 seed cultures and after 4 or 12 h of biofilm growth could be obtained at high yields and in an intact state. In contrast, samples collected from the 24 and 48 h biofilms demonstrated substantial and escalating levels of total RNA degradation (Figure S2). The levels of degraded RNA were consistent with the increasing numbers of dead bacteria observed by transmission electron microscopy at these time points (Figure S1). Thus this suggested that the degraded RNA was most likely isolated from the dead or dying pneumococci found within these biofilms.

Despite degradation, sufficient amounts of RNA could be isolated from the 24 and 48 h time points for efficient Cy3 and Cy5 labeling and subsequent microarray gene expression analyses using *S. pneumoniae* version 8 microarrays obtained from the NIAID Pathogen Functional Genomics Research Center (http://pfgrc.org). Nonetheless, to ensure that our microarray results were robust we processed 2–3 (3 replicates for 4 and 12 h time points, 2 replicates for 24 and 48 h time points) independent RNA samples for each time point (biological replicates) and performed dye-flipped hybridizations (technical replicates) for each sample, except for the 48 h time point where no technical replicates could be performed due to limiting amounts of RNA material. Stringent rules for hybridized signal strength and required minimum number of usable data points per time point were applied prior to the calculation of average expression levels and *P*-values (see methods). Thus in many instances, and in particular at later time points, data on transcript levels of some pneumococcal genes are not presented (labeled as “NA” in Tables S1, S2, and S3).

During transition from planktonic to biofilm growth we observed changes in TIGR4 gene transcription that encompassed almost all aspects of pneumococcal cell biology. The majority of genes with differential expression were down-regulated with significantly lower RNA levels for 40, 62, 32, and 62 genes at 4, 12, 24, and 48 h, respectively (Table S1). In contrast, 16, 40, 14, and 8 genes were determined to have enhanced transcription, at the same time points, respectively (Table S2). In total, a surprisingly small number of genes were observed to have altered transcription during biofilm growth. When compared to planktonic culture, 6.2% of the 1674 TIGR4 genes tested for by the microarray were determined to be significantly altered after 12 h of biofilm culture, the time point with the greatest number of differentially expressed genes. A complete list of the genes spotted on the microarray and their expression during bioflm growth is provided through the Gene Expression Omnibus (GEO, http://www.ncbi.nlm.nih.gov/geo/) accession number GSE26976. Validation of the microarray results was also performed by qRT-PCR (Figure S3).

Consistent with the concept that biofilms are in a quiescent state [38], 32 genes encoding either ribosomal proteins or translation initiation and elongation factors were down-regulated during biofilm growth. This was accompanied by a reduction in 8 genes encoding the ATP synthase machinery (*SP_ 1506- SP_1514*) and 9 other genes, including the Fab operon, involved in fatty acid metabolism and phospholipid biosynthesis (*SP_0415-SP_0427*). Other indicators that biofilm bacteria were in an inert state included decreased expression of the cell division gene *ftsZ* (*SP_1666*), decreased expression of assorted Sec pre-protein translocase components (*SP_0230, SP_1702,SP_2029*), and reduced expression of over 32 conserved hypothetical or hypothetical proteins. Despite an established role for capsule as a major component of the EPM [39], 8 of the genes encoded within the CPS cassette (*SP_0346-SP_0360*) were down-regulated during biofilm growth. Other virulence determinants with decreased expression included the type I pilus ancillary protein RrgC (*SP_0464*), pneumolysin (*SP_1923*), and choline binding protein PcpA (*SP_2136*).

In contrast, Pneumococcal serine-rich repeat protein (PsrP; *SP_1772*) was the only established virulence determinant with enhanced expression during mature biofilm growth. PsrP is a host cell and intra-species bacterial adhesin previously shown to contribute to biofilm formation. PsrP is encoded within the pathogenicity island p*srP-secY2A2* (*SP_1755-SP_1772*) along with 10 glycosyltransferases and 7 components of an alternate Sec translocase [11], [26], [40]. These accessory genes are putatively responsible for PsrP glycosylation and transport and, along with *psrP,* were significantly up-regulated during biofilm growth [41], [42], [43]. Not surprisingly, genes encoding stress-related chaperonins and proteases were also enhanced. These included genes encoding GroEL (*SP_1906*), Class I heat shock proteins (*SP_0516-SP_0517*), a member of the universal stress protein family (*SP_1996*), thioredoxin (*SP_1000*) and 2 Clp proteases (*SP_0338, SP_0820*). Three genes involved in the high affinity phosphate transport system were also elevated (*SP_2084-SP_2088*), as were 3 genes involved in glycogen synthesis (*SP_1121-SP_1123*). Interestingly, genes encoding transposases (*SP_0392, SP_0814, SP_0850, SP_1485, SP_1593, SP_1613*) were also up-regulated suggesting they are sensitive and responsive to physiological stress. Finally, 18 hypothetical proteins were also enhanced, among which SP_1793 was found to be up-regulated as high as 45-fold.

To verify our microarray results we performed qRT-PCR on 19 genes identified by the microarray as either down, no change, or up-regulated using RNA from each biological replicate (See methods; Table S3). We then determined the correlation coefficient between our microarray data with the results from the qRT-PCR. A strong positive correlation was observed with the correlation coefficients ranging from 0.66 to 0.94, except for a single 4 h replicate 1 at 0.59. Thus transcript levels as determined by microarray were reliable. Included among the genes tested by qRT-PCR were *pspA* (*SP_0117*) *and spxB* (*SP_0730*) that encode proteins responsible for complement resistance and production of hydrogen peroxide, respectively, and were confirmed as unchanged [44], [45]. *ply* that encodes pneumolysin that was confirmed as reduced. Finally, *psrP*, the Clp protease *SP_0338,* and *SP_1793,* which were all enhanced. Unexpectedly *cbpA* (*SP_2190*), which encodes the pneumococcal adhesin Choline binding protein A (CbpA) [46], [47], [48], and *nanA* (*SP_1693*), the gene encoding neuraminidase A [49], were up-regulated during biofilm growth as measured by qRT-PCR. Likewise, *rlrA* (SP_0461) and *rrgA* (SP_0462) the type I pilus transcriptional regulator and the pilus adhesin protein, respectively, were down-regulated [50], [51]. Presumably, the increased sensitivity of PCR versus detection of hybridized labeled cDNA during microarray analyses explains the uncovering of these additional differentially regulated genes by qRT-PCR.

In summation, the microarray data was in agreement with studies that suggest biofilm bacteria are quiescent. Furthermore, these studies suggest that bacteria within a mature biofilm: 1) produce less capsular polysaccharide, 2) are undergoing considerable physiological stress, and 3) with exception to PsrP and possibly CbpA and Neuraminidase A, have reduced virulence determinant expression. Notably, as determined by the unchanged expression of genes involved with competence, we did not observe that pneumococci within biofilms were in a competent state. This was consistent with findings reported by Trappetti *et al.*, showing that deletion of ComD, which senses competence stimulating peptide, had no effect on pneumococcal biofilm formation in a continuous flow-through reactor [52].

### Reduced capsule production by biofilm pneumococci

**Figure 4:**
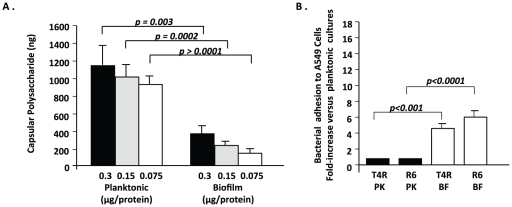
Modifications of polysaccharide capsule during biofilm growth contribute to hyper-adhesive phenotype. (A) Direct ELISA technique, using sera to type 4 polysaccharide capsule, comparing the amount of capsule present in three dilutions of whole cell lysates from planktonic or biofilm-derived bacteria. (B). Bacterial adhesion assay comparing planktonic and biofilm cultures of T4R and R6, an unencapsulated derivative of TIGR4 and serotype 2 respectively, to A549 cells. Values are expressed as fold increase adhesion relative to the planktonic counterparts. Statistical analysis was performed using a two-tailed Student's *t-*test.

Since deletion of capsule enhances bacterial adhesion but results in a complete loss of virulence [53], and because we observed a similar phenotype with biofilm pneumococci, we used a direct ELISA approach to measure total levels of CPS in TIGR4 cultures and determined that planktonic bacteria had a 3 to 5-fold greater amount of capsular polysaccharide than their biofilm derivatives (Figure 4A). We subsequently tested the ability of T4R, an unencapsulated derivative of TIGR4, and R6, an unrelated and un-encapsulated serotype 2 derivative that naturally lacks PsrP, to adhere to cells following either planktonic or biofilm culture (Figure 4B) [28], [54]. For both strains tested, a less dramatic but persistent biofilm hyper-adhesive phenotype was observed, indicating that capsule levels played an important, but not complete, role in the observed hyper-adhesive phenotype. Thus, we confirmed that the capsule is down-regulated during biofilm growth and that its reduction most likely contributed to, but was not solely responsible for, the observed hyper-adhesive state.

### Biofilm formation selects for the transparent phenotype

*S. pneumoniae* undergoes phase variation, alternating between a 1) transparent, low-capsule and high teichoic acid phase, and 2) an opaque, high-capsule and low teichoic acid state [34]. During nasopharyngeal colonization the majority of pneumococci are of the transparent phenotype, which has an enhanced ability to bind to host cells. This is due to reduced capsule, but also an increased amount of surface exposed ChoP, which binds to the host protein platelet-activating factor receptor (PAFr) on epithelial cells [27]. Importantly, C-reactive protein binds to bacterial ChoP, opsonizing the bacteria and promoting its phagocytosis [55].

**Figure 5:**
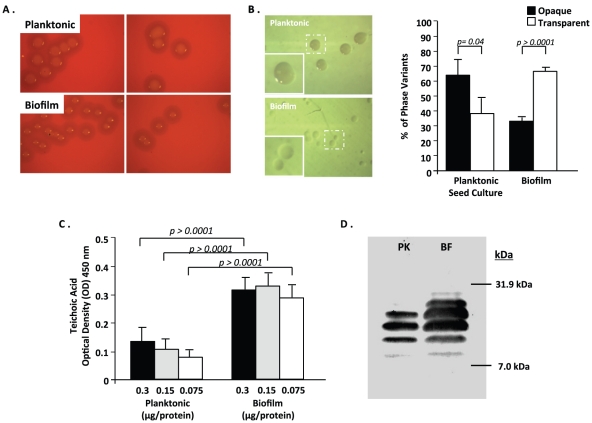
Selection for the transparent phenotype occurs during biofilm growth. (A) Representative images (n = 6) of individual bacterial colonies grown from either seed cultures (planktonic) or after 48 h of biofilm growth on blood agar plates. (B) Phase variation during biofilm growth as visualized by oblique, transmitted illumination of the planktonic culture and biofilm-derived bacteria. Percentage of phase variants in planktonic and biofilm cultures as determined by random counts of >100 individual bacterial colonies. (C) Relative levels of phosphorylcholine (ChoP) measured in whole cell lysates of planktonic or biofilm-derived bacteria were measured by Direct ELISA and by western blot analysis using monoclonal antibodies to ChoP (TEPC-15) (D) Immunoblot analysis of teichoic acids were performed using whole cell lysates of planktonic and biofilm-derived bacteria separated on 15% SDS-PAGE (E).

We observed that colonies on plates grown from biofilm isolated pneumococci were consistently smaller than those used to seed the biofilm reactor. Upon close examination by trans-oblique illumination they were determined to more frequently belong to the transparent phenotype (Figure 5A,B). We determined that 69% of pneumococci isolated from a mature biofilm were transparent. In contrast, pneumococci in the cultures used to inoculate the biofilm reactor were predominantly of the opaque phenotype (63% opaque). We confirmed the transparent phenotype by measuring teichoic acid levels. Using direct ELISA to calculate relative levels of teichoic acid in cell lysates, we observed a 3–4 fold increase in optical density of the developed ELISA for biofilm cultures (Figure 5C). This was confirmed by immunoblot for teichoic acids using TEPC-15, a mouse IgA monoclonal against ChoP (Figure 5D). Not only was a difference observed in the total amount of teichoic acid for biofilm pneumococci, but two additional, higher molecular weight teichoic acid polymers were also observed for biofilm cultures. Presence of these additional bands was consistent with published studies contrasting the two phenotypes [34].

### Biofilm pneumococci modulate pneumolysin, PsrP, and CbpA production

**Figure 6:**
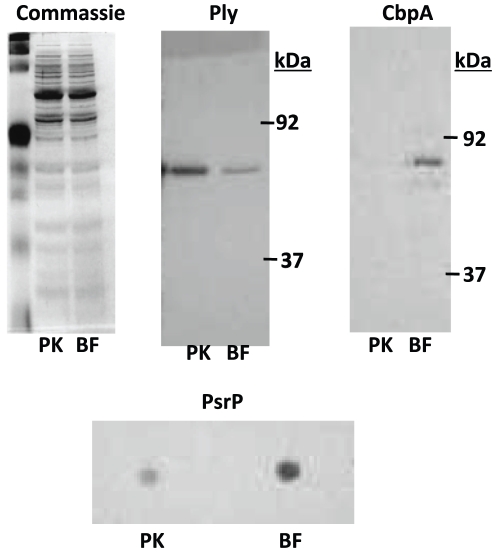
Pneumococcal modulate pneumolysin, CbpA, and PsrP during biofilm growth. Immunoblots comparing protein expression in whole cell lysates of planktonic and biofilm-derived bacteria. Whole cell lysates (5 µg) were confirmed to be equally loaded by Coomassie brilliant blue staining. Membranes were probed with antisera to pneumolysin (Ply), choline binding protein A (CbpA), and the pneumococcal serine-rich repeat protein (PsrP). For PsrP immunodot blot was used because PsrP is glycosylated and separates at a molecular weight of >4000 kDa, thus it does not enter SDS-PAGE gels normally used to separate proteins [26].

Following SDS-PAGE separation of equal protein amounts, we confirmed decreased production of pneumolysin by biofilm pneumococci as well as increased production of the bacterial adhesin CbpA which binds to laminin receptor and has been shown to be enhanced during the transparent phenotype (Figure 6) [27], [34], [56]. Immunodot blot was used to confirm enhanced PsrP production (Figure 6). Immunodot blot was used because PsrP is glycosylated and separates at a molecular weight of >4000 kDa, thus it does not readily enter the SDS-PAGE gels normally used to separate proteins [26]. Thus we confirmed changes increased biofilm production of three adhesins and reduced production of the toxin pneumolysin.

As PsrP binds to surface-exposed Cytokeratin 10, which is present on A549 cells but absent in Detroit 562 cells [26], we sought to determine the sole contribution of PsrP to the hyper-adhesive biofilm phenotype. To do this, we tested the ability of biofilm and planktonic cultures of T4 *ΔpsrP*, a PsrP deficient mutant, to adhere to these cells (Figure 3A–B). As expected, planktonic T4 *ΔpsrP* failed to adhere A549 cells but bound normally to Detroit 562 cells. Importantly, biofilm-derived T4 *ΔpsrP* still adhered to A549 and Detroit cells at levels 4 to7-fold greater than its planktonic counterparts, indicating that other factors including, but possibly not limited to, ChoP and CbpA were involved in adhesion. In support of this notion, the serotype 14 clinical isolate used in Figure panel 3B, which naturally lacks *psrP* (i.e. as determined by comparative genomic hybridization [40]), also showed enhanced biofilm-mediated adhesion.

### Planktonic growth of biofilm pneumococci partially restores virulence

Finally, we sought to determine the durability of the observed biofilm phenotype. We did this by testing the virulence of planktonic pneumococci derived from a mature biofilm sample. Following intranasal and intraperitoneal challenge, biofilm-derived planktonic pneumococci remained attenuated colonizing the nasopharynx normally but being unable to enter the bloodstream or rapidly kill mice, respectively (Figure 2A, 2C). In contrast, following intratracheal challenge, biofilm-derived planktonic pneumococci were able to establish pneumonia and present in the lungs at bacterial titers equivalent to mice challenged with stock planktonic bacteria. Interestingly, biofilm-derived planktonic pneumococci remained unable to enter the bloodstream (Figure 2B), suggesting that they had an intermediate phenotype that specifically affected translocation into the bloodstream or survival therein.

## Discussion

Intranasal and intratracheal challenge of mice with disrupted mature biofilms allowed us to directly test whether pneumococci within biofilms were in a virulent state and modeled the aspiration of bacterial aggregates that might be present in mucosal secretions from the nasopharynx or that which might be introduced during intubation [57], [58]. Our results indicate that pneumococci within biofilms are highly suited for attachment to mucosal epithelial cells, but as a result are avirulent. This was unexpected as the formation of biofilms has been suggested to be a pivotal event to numerous infectious diseases [59]. One important limitation of this study is that this approach does not examine the pathogenic potential of biofilms that form *in vivo* and *de novo*, such as within the nasopharynx during normal colonization. Thus there is the possibility that *in vivo* biofilms might act differently.

Based on our experimental results, the hyper-adhesive phenotype of biofilm pneumococci could be attributed to: i) reduced capsule which exposes bacterial surface proteins [53], ii) selection for the transparent phenotype which carries greater amounts of ChoP that binds to the host-protein PAFr [60], iii) enhanced production of CbpA which binds to Laminin receptor [20], as well as PsrP, which binds to Keratin 10 [26]. Unconfirmed by protein analysis, but possibly also contributing to the hyper-adhesive phenotype, we observed increased expression of the gene encoding Neuraminidase A by qRT-PCR, which has been shown to enhance bacterial adhesion by cleaving sialic acid moieties and thereby exposing cryptic ligands on the host-cell surface [61]. The attenuated phenotype of biofilm pneumococci could be attributed to: i) a reduced metabolic rate that would delay its ability to respond to stressors in a novel host-environment, ii) enhanced ChoP, which would enhance opsonization by C-reactive protein [55], iii) reduced capsule, which would also facilitate phagocytosis [34], iv) a reduction in pneumolysin production [25], v) reduced PcpA and possibly type I pilus [62]. While a reduction in capsule and pneumolysin expression along with enhanced neuraminidase has been shown for *S. pneumoniae* biofilms [14], [15], [63], ours is the first study to suggest they act collectively to dramatically impact the ability of biofilm pneumococci to progress from the nasopharynx and cause invasive disease; in particular bloodstream infections. Thus, implying that pneumococci within biofilms do not directly contribute to the development of invasive disease.

Using TEM to examine pneumococci within a mature biofilm structure we were surprised to determine that only a small percentage of the mature biofilm was composed of electron dense and presumably viable pneumococci. A finding that suggests robust pneumococcal biofilm formation occurred through the accumulation of dead pneumococci. Most recently, Trappetti *et al.* have shown that it is the opaque phase variant of *S. pneumoniae* that is responsible for formation of the EPM and not the transparent. As our biofilms contained both transparent and opaque *S. pneumoniae*, the opaque variant most likely accounts for the EPM we detected by electron microscopy. Furthermore, and in contrast to our *in vivo* findings, Trappetti *et al*. observed that opaque but not transparent biofilm-derived pneumococci, were able to translocate from the nasopharynx to the lungs and brain of mice [19]. One possible explanation for this discrepancy in results is that the transparent pneumococci present in our biofilms facilitated the opsonophagocytosis of the attached opaque bacteria. This would suggest that naturally occurring mixed biofilms are avirulent. Alternatively, is our use of a continuous flow reactor for mature biofilm development; Trappetti *et al*. used a static biofilm model. In separate studies both Trappetti *et al.* and ourselves found that that the use of different biofilm models resulted in variable phenotypes [11], [52]. Finally, is our use of the TIGR4 strain of *S. pneumoniae* whereas Trappetti *et al.* used a 19F clinical isolate.

Despite our observation of considerable EPM surrounding the electron dense and presumably viable bacteria, biofilm pneumococci were determined to be hyper-adhesive, suggesting that in addition to a loss in capsule and increased ChoP, CbpA, and PsrP protein levels by pneumococci, the EPM may also contain adhesive elements. This possibility is also supported by findings by Trappetti *et al.*, which determined that opaque sessile (i.e biofilm) pneumococci adhere to A549 and Detroit 562 cells better than transparent sessile pneumococci. The latter was unexpected as the transparent phenotype is associated with increased expression of CbpA and ChoP and transparent planktonic pneumococci have been shown to adhere to cells in an enhanced manner [24], [34], [64]. It is for this latter reason that we believe mice challenged with biofilm-derived planktonic pneumococci, which would be mostly transparent, developed pneumonia but were unable to cause bloodstream infection. Importantly, our observation of enhanced biofilm adhesion by numerous strains and enhanced PsrP, ChoP, and CbpA production indicates that the hyper-adhesive phenotype is a pan-pneumococcal biofilm property that is multi-factorial, involving numerous components along with the production of EPM.

In an effort to develop a working model that coalesces the published data with our own, we propose that the selective death of opaque pneumococci might be occurring during biofilm formation. Opaque cell death would provide a mechanism for the release of DNA and other components that are known make up the EPM. It would also provide an explanation for the high numbers of dead bacteria observed in our biofilms as well as our recovery of predominantly transparent pneumococci from mature biofilms. In context of *in vivo* transmission our model implies that the opaque variant would be responsible for formation of the EPM in the nasopharynx and thereby confer *in vivo* persistence, whereas the transparent variant, which is better able to attach to cells and colonize naïve animals [34], would remain available in greater numbers for spread to the next host and gain from the enhanced adhesive capacity of the surrounding EPM. In the next host, partial reversion to the opaque variant would t also be necessary to reform a biofilm. Thus, studies are warranted to ascertain if differential cell death dependent on phase-variation occurs within biofilms and to test its impact on transmission.

The observed tolerance of biofilm pneumococci to antimicrobials was in agreement with previously published studies [29], moreover, was indicative that we were in fact examining mature biofilms. Importantly, biofilm pneumococci remained susceptible to cell wall acting antimicrobials suggesting that maintenance of the cell wall remained a critical function during the quiescent state. Ours is the most comprehensive analysis of pneumococcal gene expression during biofilm growth to date. However, our gene expression data reflects the biases of our biofilm model which includes biofilm-related changes in the ratio of opaque and transparent pneumococci as well as increasing amounts of remnant mRNA from dead bacteria. This most likely explains why our microarray results do not exactly match previous studies that explore differences between opaque and transparent pneumococci [19], [65].

We determined that biofilm bacteria down-regulated >50 genes involved in protein translation, the ATP synthase machinery, fatty acid metabolism, phospholipid synthesis, and replication. A strong reduction in capsule operon cassette expression was observed consistent with a previous study by Moscoso *et al.*, which showed that *cps3A*, the first gene in the capsule operon cassette was down-regulated during biofilm production [63]. Previously, for serotype 3 strains, a non-phase variable deletion within the capsule operon cassette resulting in a rough mutant has been shown to occur and contribute towards biofilm formation [66]. A reduction in capsule would serve to expose surface components such as adhesins and facilitate attachment. This notion is supported by our previous findings with PsrP, where a version of the protein unable to extend past the capsule layer failed to mediate adhesion, as well studies as completed by Munoz-Elias *et al.*, that found use of an unencapsulated strain facilitated the identification of genes involved in biofilm formation *in vitro* [17], [26]. Concomitantly, a reduction in capsule would reduce the virulence potential of individual pneumococci.

As indicated, the observed reduction in the physiological state of bacteria may also contribute to their attenuated phenotype. Metabolically inert bacteria would take longer to adapt to hostile host environments such as the lower respiratory tract and produce the necessary determinants required for survival such as pneumolysin. Along this line, our observation that biofilm pneumococci down-regulate pneumolysin allows for speculation that biofilm pneumococci stop producing factors that elicit a strong inflammatory response during biofilm formation within the nasopharynx. Presumably, this would promote long-term colonization by modulating the immune response. This concept is supported by the finding that invasive serotypes of *S. pneumoniae* colonize the nasopharynx for a shorter duration than non-invasive serotypes [67]. The reduction in pneumolysin and PcpA levels during biofilm growth also suggests that immunization with pneumolysin or PcpA would also have a modest effect against colonization but might still protect against disseminated (i.e. planktonic) disease. In contrast, antibodies against CbpA and PsrP, which are up-regulated during biofilm growth, might deter nasopharyngeal colonization and thereby promote species replacement, such as with *Staphylococcus aureus*, in immunized individuals. As such the differential production of protein vaccine candidates during biofilm versus planktonic growth should be an important consideration in the design of any future protein vaccine against *S. pneumoniae* or other bacterial pathogens [68]. Of note this concept is consistent with findings by Oggioni *et al.,* showing altered pneumococcal virulence gene expression occurred during sessile bacterial growth on fixed surfaces versus planktonic [14].

Finally, the enhanced expression of *psrP* and its accessory proteins was in agreement with our previous studies that showed PsrP contributes to robust biofilm formation [26]. While unconfirmed microarray and qRT-PCR data implies that the type I pilus might be down regulated during biofilm production; this would be surprising as the pilus of Group A Streptococci and Group B Streptococci have been shown to play an important role in biofilm formation [69], [70]. Of note, microarrays did not reveal enhanced *cbpA* expression, however, it was determined by qRT-PCR and immunoblot that CbpA levels were increased. This emphasizes the necessity for validation of RNA data with protein studies and raises the possibility that other determinants are also altered. Thus a proteomics approach is warranted to address this gap. Of note, our microarray findings are in stark contrast to those by Allegrucci *et al.*, who found a dramatic increase in the number of detectable biofilm proteins when examining 2-dimensional gels of a serotype 3 isolate [13]. A possible explanation for this discrepancy is the accumulation of dead bacteria and their proteins in a mature biofilm. This would be an inseparable and confounding factor in any proteomic analysis of mature biofilms.

In summary, we observed a dramatic enhancement in the ability of biofilm pneumococci to attach to host cells as well as a dramatic reduction in their ability to cause invasive disease. Notably, biofilm pneumococci colonized the nasopharynx normally. As the vast majority of *S. pneumoniae* do not cause invasive disease, it is most likely that these biofilm related changes occur so as to facilitate long-term colonization of the nasopharynx rather than promote development of invasive disease. Therefore, and based on the available information, we suggest that the ability to form robust biofilms is not required for virulence, but instead contributes towards long-term colonization and transmission of the pneumococcus.

## Supporting Information

Figure S1 **Quantification of viable and dead cells during mature pneumococcal biofilm development.** (A) Representative Transmission Electron Microscopy (TEM) images of biofilm bacteria at designated time points; white bars represent 2 microns. Note that viable cells are electron dense, while dead cells appear as lighter images or “ghosts”. (B) Enumeration of viable and dead cells during mature biofilm development. Percentages of viable and dead pneumococci as determined by cell counts from six images per indicated time point.
(EPS)

Figure S2 **Assessment of the quality of isolated bacterial RNA from mature pneumococcal biofilms.** (A) Formaldehyde gels demonstrating the quality of RNA samples taken from *S. pneumoniae* biofilms grown under once-through conditions at the designated time points. (B) RNA profiles of samples collected from biofilms above using the Agilent 2100 Bioanalyzer. Note the considerable amount of degradation of RNAs at the later time points.
(EPS)

Figure S3 **Validation of microarray studies.** RNA levels obtained with microarrays (X-axis) and qRT-PCR (y-axis) are in good agreement. The qRT-PCR ΔCt values (Livak and Schmittgen, 2001) (y-axis) are compared to the log2 transformation of microarray query/reference ratios (x-axis) for each biological replicate (2–3) of each time point (4–48 h). Log2 values are used to obtain a linear correlation. Strong correlation coefficients are observed, ranging from 0.66 to 0.94, except for at the 4 h replicate 1 at 0.59.
(EPS)

Table S1 **Genes with significantly decreased expression in at least one time point during biofilm growth.**
(DOCX)

Table S2 **Genes with significantly increased expression in at least one time point during biofilm growth.**
(DOCX)

Table S3 **Relative expression of** ***S. pneumoniae*** **genes following biofilm growth versus planktonic cultures as determined by microarray and qRT-PCR ΔCT values.**
(DOCX)
