# Supplementary material for: Streptococcus pneumoniae in Biofilms Are Unable to Cause Invasive Disease Due to Altered Virulence Determinant Production
Source: PLoS One. 2011 Dec 8;6(12):e28738. doi: 10.1371/journal.pone.0028738 (PMC3234282; doi:10.1371/journal.pone.0028738)
Supplement: Table S1 — Genes with significantly decreased expression in at least one time point during biofilm growth. (DOCX) [file pone.0028738.s004.docx]

| **Table S1.** Genes with significantly decreased expression in at least one time point during biofilm growth* | | | | | | | | | | | | |
| --- | --- | --- | --- | --- | --- | --- | --- | --- | --- | --- | --- | --- |
| **TIGR4** | **Annotation** | **4 hour** | ***p* value** |  | **12 hour** | ***p* value** |  | **24 hour** | ***p* value** |  | **48 hour** | ***p* value** |
| SP_0024 | conserved hypothetical protein | **0.43** | <0.0001 |  | 0.88 | 1.0000 |  | **0.37** | 0.0001 |  | 0.71 | 1.0000 |
| SP_0025 | conserved hypothetical protein | **0.40** | 0.0001 |  | 0.74 | 1.0000 |  | **0.37** | 0.0047 |  | 0.84 | 1.0000 |
| SP_0056 | adenylosuccinate lyase | **0.53** | 0.0076 |  | 0.67 | 1.0000 |  | 0.53 | 0.6501 |  | 0.67 | 1.0000 |
| SP_0095 | conserved hypothetical protein | **0.38** | <0.0001 |  | **0.47** | 0.0002 |  | 0.28 | 0.0211 |  | **0.32** | 0.0012 |
| SP_0121 | conserved hypothetical protein | 0.61 | 0.0826 |  | **0.53** | 0.0069 |  | 0.47 | 0.4313 |  | 0.44 | 0.7198 |
| SP_0122 | conserved domain protein | 0.67 | 1.0000 |  | 0.52 | 0.1549 |  | **0.30** | 0.0001 |  | 0.33 | 1.0000 |
| SP_0178 | riboflavin biosynthesis protein RibD | 1.33 | 1.0000 |  | **0.44** | 0.0017 |  | 0.90 | 1.0000 |  | 0.50 | 0.5467 |
| SP_0202 | anaerobic ribonucleoside-triphosphate reductase | **0.39** | 0.0002 |  | 0.55 | 1.0000 |  | 0.43 | 0.0842 |  | 1.77 | 1.0000 |
| SP_0208 | ribosomal protein S10 | 0.82 | 1.0000 |  | 0.81 | 1.0000 |  | 0.49 | 1.0000 |  | **0.37** | 0.0001 |
| SP_0209 | 50S Ribosomal protein L3 | 0.99 | 1.0000 |  | 0.74 | 1.0000 |  | 0.43 | 1.0000 |  | **0.23** | 0.0007 |
| SP_0210 | 50S ribosomal protein L4 | 0.73 | 1.0000 |  | 0.73 | 1.0000 |  | 0.51 | 1.0000 |  | **0.31** | 0.0004 |
| SP_0211 | ribosomal protein L23 | 0.68 | 1.0000 |  | 0.68 | 1.0000 |  | 0.48 | 1.0000 |  | **0.22** | <0.0001 |
| SP_0213 | 30S ribosomal protein S19 | 0.55 | 1.0000 |  | 0.66 | 1.0000 |  | 0.55 | 1.0000 |  | **0.19** | <0.0001 |
| SP_0215 | ribosomal protein S3 | 0.58 | 0.5476 |  | 0.71 | 1.0000 |  | 0.49 | 1.0000 |  | **0.29** | 0.0022 |
| SP_0218 | 30S ribosomal protein S17 | 0.55 | 0.3318 |  | 0.70 | 1.0000 |  | 0.47 | 1.0000 |  | **0.27** | <0.0001 |
| SP_0219 | 50S ribosomal protein L14 | 0.75 | 1.0000 |  | 0.98 | 1.0000 |  | 0.26 | 0.5822 |  | **0.30** | 0.0001 |
| SP_0220 | ribosomal protein L24 | 0.60 | 0.0757 |  | 0.90 | 1.0000 |  | 0.35 | 1.0000 |  | **0.27** | 0.0020 |
| SP_0221 | 50S Ribosomal protein L5 | 0.64 | 1.0000 |  | 0.76 | 1.0000 |  | 0.45 | 1.0000 |  | **0.17** | <0.0001 |
| SP_0222 | 30S Ribosomal protein S14 | 0.60 | 1.0000 |  | 0.89 | 1.0000 |  | 0.50 | 1.0000 |  | **0.30** | 0.0002 |
| SP_0225 | 50S Ribosomal protein L6 | 0.51 | 0.4133 |  | 0.81 | 1.0000 |  | 0.57 | 1.0000 |  | **0.19** | 0.0003 |
| SP_0226 | ribosomal protein L18 | 0.51 | 1.0000 |  | 0.80 | 1.0000 |  | 0.49 | 1.0000 |  | **0.31** | 0.0003 |
| SP_0227 | 30S ribosomal protein S5 | 0.58 | 0.9772 |  | 0.96 | 1.0000 |  | 0.53 | 1.0000 |  | **0.22** | <0.0001 |
| SP_0228 | 50S Ribosomal protein L30 | 0.52 | 0.0844 |  | 0.89 | 1.0000 |  | 0.58 | 1.0000 |  | **0.15** | <0.0001 |
| SP_0229 | 50S ribosomal protein L15 | 0.54 | 1.0000 |  | 0.73 | 1.0000 |  | 0.50 | 1.0000 |  | **0.25** | 0.0004 |
| SP_0230 | preprotein translocase, SecY subunit | 0.54 | 0.0529 |  | 0.82 | 1.0000 |  | 0.53 | 1.0000 |  | **0.21** | 0.0027 |
| SP_0235 | ribosomal protein S11 | 1.19 | 1.0000 |  | 0.72 | 1.0000 |  | NA | NA |  | **0.08** | 0.0097 |
| SP_0238 | conserved hypothetical protein | **0.51** | 0.0040 |  | 0.42 | 0.0147 |  | 0.32 | 0.6061 |  | 1.30 | 0.7454 |
| SP_0239 | conserved hypothetical protein | 0.65 | 1.0000 |  | 0.46 | 0.0313 |  | **0.41** | 0.0021 |  | 1.17 | 1.0000 |
| SP_0271 | ribosomal protein S12 | 0.76 | 1.0000 |  | 0.63 | 1.0000 |  | 0.48 | 1.0000 |  | **0.24** | 0.0016 |
| SP_0273 | translation elongation factor G | 0.76 | 1.0000 |  | 0.62 | 1.0000 |  | 0.52 | 1.0000 |  | **0.37** | 0.0027 |
| SP_0283 | Phosphotransferase system, mannose-specific EIIC | **0.32** | 0.0012 |  | 0.50 | 1.0000 |  | 0.45 | 1.0000 |  | 0.28 | 0.0813 |
| SP_0284 | PTS system, mannose-specific IIAB components | **0.41** | 0.0004 |  | 0.64 | 1.0000 |  | 0.57 | 1.0000 |  | 0.24 | 0.0537 |
| SP_0290 | dihydrofolate synthetase | 0.64 | 1.0000 |  | 1.00 | 0.9989 |  | **0.63** | 0.0022 |  | 1.34 | 1.0000 |
| SP_0294 | 50S ribosomal protein L13 | 0.67 | 1.0000 |  | 0.46 | 0.2836 |  | 0.36 | 0.5792 |  | **0.19** | 0.0010 |
| SP_0295 | 30S ribosomal protein S9 | 0.58 | 1.0000 |  | 0.42 | 0.1906 |  | 0.47 | 1.0000 |  | **0.25** | 0.0007 |
| SP_0341 | conserved hypothetical protein | 0.75 | 1.0000 |  | 0.55 | 0.1342 |  | **0.35** | 0.0033 |  | 1.43 | 1.0000 |
| SP_0346 | capsular polysaccharide biosynthesis protein Cps4A | **0.46** | 0.0009 |  | 0.64 | 1.0000 |  | 0.46 | 1.0000 |  | 0.48 | 0.3035 |
| SP_0347 | capsular polysaccharide biosynthesis protein Cps4B | **0.49** | 0.0012 |  | 0.59 | 0.0189 |  | 0.52 | 0.8448 |  | 0.60 | 1.0000 |
| SP_0350 | capsular polysaccharide biosynthesis protein Cps4E | **0.29** | 0.0001 |  | 0.45 | 0.0766 |  | 0.38 | 0.4262 |  | 0.37 | 0.0381 |
| SP_0352 | capsular polysaccharide biosynthesis protein Cps4G | **0.35** | 0.0095 |  | 0.41 | 0.0152 |  | 0.38 | 1.0000 |  | **0.30** | 0.0077 |
| SP_0355 | hypothetical protein | 0.36 | 0.2543 |  | 0.47 | 0.2463 |  | 0.34 | 1.0000 |  | **0.30** | 0.0010 |
| SP_0356 | putative polysaccharide transporter | 0.38 | 1.0000 |  | 0.48 | 0.6168 |  | 0.32 | 0.4480 |  | **0.34** | 0.0001 |
| SP_0357 | UDP-N-acetylglucosamine-2-epimerase | **0.40** | 0.0054 |  | 0.55 | 0.1186 |  | 0.44 | 0.4987 |  | 0.41 | 0.0883 |
| SP_0360 | UDP-N-acetylglucosamine 2-epimerase | 0.41 | 0.0102 |  | 0.57 | 0.0282 |  | 0.53 | 1.0000 |  | **0.26** | 0.0009 |
| SP_0400 | trigger factor | 0.55 | 1.0000 |  | **0.39** | 0.0049 |  | 0.45 | 0.6186 |  | 0.35 | 0.0274 |
| SP_0411 | seryl-tRNA synthetase | 0.75 | 1.0000 |  | 0.69 | 1.0000 |  | **0.45** | <0.0001 |  | 0.40 | 0.1319 |
| SP_0412 | conserved hypothetical protein | 0.72 | 1.0000 |  | 0.60 | 0.8615 |  | **0.54** | 0.0009 |  | 0.83 | 1.0000 |
| SP_0415 | enoyl-CoA hydratase/isomerase family protein | **0.29** | 0.0009 |  | **0.46** | 0.0086 |  | 0.35 | 0.0475 |  | **0.25** | 0.0035 |
| SP_0417 | 3-oxoacyl-(acyl-carrier-protein) synthase III | **0.38** | 0.0022 |  | 0.45 | 0.0297 |  | 0.41 | 0.2402 |  | **0.33** | 0.0024 |
| SP_0419 | Enoyl-acyl carrier protein(ACP) reductase. | **0.25** | <0.0001 |  | **0.41** | 0.0046 |  | 0.44 | 1.0000 |  | **0.22** | 0.0073 |
| SP_0420 | Malonyl acyl carrier protein transacylase | **0.25** | <0.0001 |  | **0.31** | <0.0001 |  | **0.34** | 0.0041 |  | **0.25** | 0.0006 |
| SP_0421 | 3-oxoacyl-[acyl-carrier protein] reductase | **0.32** | 0.0053 |  | **0.31** | <0.0001 |  | **0.30** | 0.0012 |  | 0.51 | 1.0000 |
| SP_0422 | 3-oxoacyl-(acyl-carrier-protein) synthase II | **0.32** | 0.0074 |  | **0.32** | 0.0009 |  | 0.34 | 0.7200 |  | 0.29 | 0.0137 |
| SP_0423 | acetyl-CoA carboxylase, bitoin carboxyl carrier protein | **0.42** | 0.0022 |  | 0.46 | 0.0248 |  | 0.41 | 0.0260 |  | 0.31 | 0.0412 |
| SP_0424 | Hydroxymyristoyl-(acyl carrier protein) dehydratase | 0.39 | 0.0462 |  | **0.36** | 0.0006 |  | 0.51 | 1.0000 |  | 0.35 | 0.0612 |
| SP_0427 | Acetyl-coenzyme A carboxylase carboxyl transferase subunit alpha | 0.60 | 0.0555 |  | **0.37** | <0.0001 |  | 0.59 | 1.0000 |  | **0.26** | 0.0010 |
| SP_0433 | Transcription termination protein | 0.78 | 1.0000 |  | **0.47** | 0.0031 |  | 0.47 | 0.2957 |  | 0.42 | 0.0585 |
| SP_0464 | pilus ancillary protein RrgC | **0.30** | 0.0004 |  | 0.46 | 1.0000 |  | 0.49 | 0.2545 |  | 0.33 | 1.0000 |
| SP_0488 | conserved hypothetical protein | 0.55 | 1.0000 |  | **0.30** | 0.0030 |  | 0.30 | 1.0000 |  | 0.66 | 1.0000 |
| SP_0489 | conserved hypothetical protein | **0.41** | 0.0009 |  | 0.48 | 0.2364 |  | **0.40** | 0.0089 |  | 0.47 | 1.0000 |
| SP_0492 | conserved hypothetical protein | **0.56** | 0.0078 |  | 0.34 | 0.0107 |  | 0.39 | 0.0492 |  | 0.61 | 1.0000 |
| SP_0493 | putative DNA-directed RNA polymerase, delta subunit | 0.49 | 0.0709 |  | **0.39** | 0.0027 |  | 0.38 | 0.0970 |  | 0.65 | 0.0615 |
| SP_0501 | Transcriptional repressor of the glutamine synthetase gene | **0.15** | <0.0001 |  | **0.19** | 0.0001 |  | **0.20** | 0.0106 |  | **0.35** | 0.0475 |
| SP_0502 | glutamine synthetase, type I | **0.22** | 0.0011 |  | **0.20** | 0.0005 |  | **0.18** | 0.0006 |  | 0.92 | 1.0000 |
| SP_0631 | 50S ribosomal protein L1 | 0.75 | 1.0000 |  | 0.46 | 0.1346 |  | 0.36 | 1.0000 |  | **0.64** | 0.0004 |
| SP_0677 | conserved hypothetical protein | 0.83 | 1.0000 |  | 0.51 | 0.0178 |  | **0.47** | 0.0053 |  | NA | NA |
| SP_0678 | conserved hypothetical protein | 0.63 | 1.0000 |  | **0.42** | 0.0002 |  | 0.36 | 1.0000 |  | 1.16 | 1.0000 |
| SP_0681 | GTP-binding protein TypA/BipA (tyrosine phosphorylated protein A). | 0.56 | 1.0000 |  | **0.44** | 0.0048 |  | 0.34 | 1.0000 |  | **0.32** | 0.0002 |
| SP_0682 | Hypothetical protein | 0.87 | 1.0000 |  | **0.49** | 0.0050 |  | 0.50 | 1.0000 |  | 0.96 | 1.0000 |
| SP_0775 | 30S ribosomal protein S16 | 0.52 | 0.1375 |  | **0.34** | <0.0001 |  | 0.35 | 1.0000 |  | 0.77 | 1.0000 |
| SP_0862 | 30S Ribosomal protein S1 | 0.44 | 0.0382 |  | 0.46 | 0.0491 |  | 0.35 | 1.0000 |  | **0.29** | 0.0001 |
| SP_0866 | hypothetical protein | 0.74 | 1.0000 |  | 0.71 | 1.0000 |  | **0.51** | 0.0018 |  | 2.02 | 1.0000 |
| SP_0867 | ABC transporter, ATP-binding protein | 0.89 | 1.0000 |  | 0.99 | 1.0000 |  | 0.61 | 1.0000 |  | **0.60** | 0.0016 |
| SP_0896 | 6-phosphofructokinase | **0.66** | 0.0089 |  | 0.68 | 1.0000 |  | 0.42 | 1.0000 |  | 0.46 | 0.5896 |
| SP_0897 | pyruvate kinase, frameshift | 0.56 | 1.0000 |  | **0.47** | 0.0022 |  | 0.37 | 1.0000 |  | 0.49 | 1.0000 |
| SP_0944 | uridylate kinase | 0.73 | 1.0000 |  | **0.65** | 0.0092 |  | 0.55 | 1.0000 |  | **0.60** | 0.0035 |
| SP_0959 | translation initiation factor IF-3 | **0.56** | 0.0086 |  | 0.59 | 1.0000 |  | 0.22 | 0.1687 |  | **0.28** | 0.0002 |
| SP_1003 | pneumococcal histidine triad protein A/B (phtA/B); SP_1174 | 0.76 | 1.0000 |  | 0.83 | 1.0000 |  | **0.51** | 0.0006 |  | 1.11 | 1.0000 |
| SP_1113 | DNA-binding protein HU | 0.75 | 1.0000 |  | **0.55** | 0.0042 |  | 0.60 | 1.0000 |  | 0.71 | 1.0000 |
| SP_1220 | L-lactate dehydrogenase | 0.86 | 1.0000 |  | **0.43** | 0.0003 |  | 0.61 | 1.0000 |  | 0.70 | 0.6684 |
| SP_1231 | flavoprotein | 0.57 | 0.1470 |  | **0.38** | <0.0001 |  | **0.38** | 0.0027 |  | 0.92 | 1.0000 |
| SP_1241 | ABC transporter, amino acid-binding protein/permease protein | **0.30** | 0.0019 |  | 0.34 | 1.0000 |  | 0.24 | 0.0228 |  | 0.19 | 0.5025 |
| SP_1275 | carbamoyl-phosphate synthase pyrimidine-specific large chain | 0.55 | 0.0618 |  | **0.45** | 0.0088 |  | 0.58 | 0.4481 |  | 1.17 | 1.0000 |
| SP_1277 | aspartate carbamoyltransferase | 0.48 | 0.0673 |  | **0.42** | 0.0028 |  | 0.47 | 0.1489 |  | 0.79 | 1.0000 |
| SP_1293 | 50S ribosomal protein L19 | 0.51 | 0.4481 |  | **0.35** | 0.0001 |  | 0.43 | 0.7802 |  | 0.80 | 1.0000 |
| SP_1299 | 50S ribosomal protein L31 | 0.62 | 1.0000 |  | **0.45** | <0.0001 |  | 0.53 | 0.6593 |  | 0.83 | 1.0000 |
| SP_1306 | glutamate dehydrogenase | 0.44 | 0.1969 |  | 0.47 | 0.0187 |  | **0.45** | 0.0078 |  | 0.53 | 1.0000 |
| SP_1355 | ribosomal protein L10 | 0.45 | 1.0000 |  | 0.44 | 0.1300 |  | 0.35 | 0.8499 |  | **0.16** | 0.0006 |
| SP_1376 | shikimate dehydrogenase | 0.85 | 1.0000 |  | 0.84 | 1.0000 |  | **0.44** | 0.0003 |  | 0.96 | 1.0000 |
| SP_1395 | Phosphate transport system regulatory protein | 0.66 | 1.0000 |  | 0.68 | 1.0000 |  | 0.55 | 1.0000 |  | **0.48** | 0.0034 |
| SP_1399 | ;putative phosphate ABC transporter, permease protein | 0.99 | 1.0000 |  | 0.74 | 1.0000 |  | 0.54 | 1.0000 |  | **0.37** | 0.0027 |
| SP_1405 | conserved hypothetical protein | 0.72 | 1.0000 |  | **0.49** | 0.0088 |  | 0.58 | 0.4867 |  | 1.67 | 1.0000 |
| SP_1465 | hypothetical protein | 0.73 | 1.0000 |  | **0.42** | 0.0100 |  | 0.36 | 0.1528 |  | **0.41** | 0.0072 |
| SP_1466 | Conserved hypothetical protein | 0.56 | 1.0000 |  | **0.35** | 0.0083 |  | 0.44 | 0.9353 |  | 0.32 | 1.0000 |
| SP_1473 | conserved hypothetical protein | 0.75 | 1.0000 |  | **0.60** | 0.0024 |  | 0.75 | 1.0000 |  | 0.78 | 1.0000 |
| SP_1491 | glycerol uptake facilitator protein, putative | 0.49 | 0.1231 |  | **0.36** | 0.0016 |  | 0.35 | 0.3594 |  | **0.24** | 0.0002 |
| SP_1498 | Phosphoglucomutase/phosphomannomutase family protein | 0.86 | 1.0000 |  | **0.59** | 0.0003 |  | 0.46 | 0.0156 |  | 0.51 | 0.5638 |
| SP_1501 | amino acid ABC transporter, ATP-binding protein | **0.57** | 0.0055 |  | 0.54 | 0.0376 |  | **0.45** | 0.0024 |  | NA | NA |
| SP_1506 | conserved hypothetical protein | 0.69 | 1.0000 |  | **0.54** | 0.0015 |  | 0.45 | 1.0000 |  | 0.85 | 1.0000 |
| SP_1507 | ATP synthase F1, epsilon chain | 0.70 | 1.0000 |  | 0.67 | 1.0000 |  | 0.70 | 1.0000 |  | **0.47** | 0.0008 |
| SP_1508 | ATP synthase F1, beta chain | 0.48 | 1.0000 |  | 0.48 | 0.1522 |  | 0.48 | 1.0000 |  | **0.48** | 0.0092 |
| SP_1509 | Proton-translocating ATPase, F1 sector, gamma-subunit | 0.43 | 0.1375 |  | **0.42** | 0.0098 |  | 0.40 | 0.2149 |  | **0.36** | 0.0017 |
| SP_1510 | ATP synthase F1, alpha chain | **0.41** | 0.0013 |  | **0.45** | <0.0001 |  | 0.37 | 0.5163 |  | 0.37 | 0.1401 |
| SP_1512 | Proton-translocating ATPase, F0 sector, subunit b | **0.41** | 0.0039 |  | **0.39** | 0.0038 |  | 0.28 | 0.0673 |  | 0.35 | 0.1605 |
| SP_1513 | ATP synthase F0, A chain | 0.34 | 0.1645 |  | 0.35 | 0.0183 |  | 0.27 | 0.2586 |  | **0.27** | 0.0036 |
| SP_1514 | ATP synthase F0, C subunit | **0.31** | 0.0004 |  | **0.36** | <0.0001 |  | 0.28 | 1.0000 |  | 0.32 | 0.2090 |
| SP_1539 | 30S Ribosomal protein S18 | 0.68 | 1.0000 |  | 0.53 | 0.7777 |  | 0.44 | 1.0000 |  | **0.35** | 0.0018 |
| SP_1540 | single-stranded DNA-binding protein (ssb) | 0.65 | 1.0000 |  | 0.56 | 0.1033 |  | 0.33 | 1.0000 |  | **0.33** | <0.0001 |
| SP_1541 | ribosomal protein S6 | 0.67 | 1.0000 |  | 0.51 | 0.0338 |  | 0.34 | 1.0000 |  | **0.27** | 0.0002 |
| SP_1544 | aspartate aminotransferase | 0.91 | 1.0000 |  | 0.70 | 1.0000 |  | **0.54** | 0.0018 |  | 0.57 | 0.6431 |
| SP_1574 | Triose phosphate isomerase | 0.57 | 0.6013 |  | **0.46** | 0.0007 |  | 0.32 | 0.0462 |  | 0.38 | 0.554 |
| SP_1586 | Conserved hypothetical protein | 0.74 | 1.0000 |  | 1.00 | 1.0000 |  | **0.48** | 0.0049 |  | 0.53 | 0.0390 |
| SP_1602 | Required for expression of the phosphonate utilization phenotype in *E. coli* | 0.71 | 1.0000 |  | **0.56** | 0.0006 |  | 0.47 | 0.4495 |  | 0.46 | 0.1248 |
| SP_1626 | 30S ribosomal protein S15 | 0.55 | 1.0000 |  | **0.31** | 0.0004 |  | 0.27 | 0.2760 |  | 0.54 | 0.5843 |
| SP_1631 | threonyl-tRNA synthetase | 0.63 | 1.0000 |  | **0.49** | 0.0023 |  | 0.59 | 1.0000 |  | 0.62 | 1.0000 |
| SP_1647 | endopeptidase O | 0.76 | 1.0000 |  | **0.58** | 0.0052 |  | 0.43 | 0.0433 |  | 0.66 | 0.1040 |
| SP_1659 | isoleucyl-tRNA synthetase | 0.78 | 1.0000 |  | **0.60** | 0.0035 |  | 0.68 | 1.0000 |  | 0.61 | 1.0000 |
| SP_1663 | Conserved hypothetical protein | 0.70 | 1.0000 |  | 0.65 | 1.0000 |  | 0.53 | 1.0000 |  | **0.55** | 0.0002 |
| SP_1666 | Cell division protein FtsZ | 0.85 | 1.0000 |  | 0.67 | 1.0000 |  | 0.58 | 1.0000 |  | **0.49** | 0.0001 |
| SP_1702 | Preprotein translocase secA subunit | 0.69 | 1.0000 |  | 0.60 | 0.5284 |  | **0.42** | 0.0004 |  | 0.71 | 1.0000 |
| SP_1737 | Hypothetical protein | 0.70 | 1.0000 |  | **0.59** | 0.0021 |  | **0.47** | 0.0005 |  | 0.58 | 1.0000 |
| SP_1738 | guanylate kinase | 0.63 | 0.5401 |  | **0.57** | 0.0051 |  | 0.45 | 0.0190 |  | **0.43** | 0.0011 |
| SP_1739 | Conserved hypothetical protein | 0.68 | 1.0000 |  | 0.54 | 0.2004 |  | **0.35** | 0.0001 |  | 0.63 | 1.0000 |
| SP_1838 | glycosyl transferase, putative | 0.71 | 1.0000 |  | **0.65** | 0.0034 |  | 0.65 | 1.0000 |  | **0.32** | 0.0017 |
| SP_1887 | ABC transporter, ATP-binding protein | 0.78 | 1.0000 |  | 0.95 | 1.0000 |  | 0.80 | 1.0000 |  | **0.30** | 0.0010 |
| SP_1888 | oligopeptide ABC transporter, ATP-binding protein AmiE | 0.64 | 1.0000 |  | 0.84 | 1.0000 |  | 0.72 | 1.0000 |  | **0.31** | 0.0028 |
| SP_1891 | oligopeptide ABC transporter, oligopeptide-binding protein AmiA | 0.64 | 1.0000 |  | 0.73 | 1.0000 |  | 0.43 | 1.0000 |  | **0.33** | 0.0036 |
| SP_1922 | conserved hypothetical protein | 0.51 | 0.5411 |  | **0.37** | <0.0001 |  | 0.46 | 0.3508 |  | 0.61 | 1.0000 |
| SP_1923 | pneumolysin | 0.35 | 0.0425 |  | **0.26** | 0.0001 |  | 0.39 | 0.2900 |  | 0.79 | 1.0000 |
| SP_1924 | Hypothetical protein | **0.32** | 0.0008 |  | **0.23** | <0.0001 |  | **0.26** | 0.0005 |  | 0.51 | 0.0358 |
| SP_1925 | hypothetical protein | 0.42 | 0.2228 |  | 0.40 | 0.8207 |  | **0.23** | 0.0005 |  | **0.35** | 0.0005 |
| SP_1926 | hypothetical protein | **0.32** | 0.0006 |  | **0.33** | <0.0001 |  | **0.30** | 0.0002 |  | 0.48 | 0.0686 |
| SP_1970 | aspartate--ammonia ligase | **0.41** | <0.0001 |  | **0.39** | 0.0001 |  | 0.32 | 0.0199 |  | **0.51** | 0.0085 |
| SP_1978 | Diaminopimelate decarboxylase | **0.61** | <0.0001 |  | **0.49** | 0.0067 |  | 0.46 | 0.8882 |  | 0.73 | 1.0000 |
| SP_2028 | phosphotyrosine protein phosphatase | 0.62 | 0.0241 |  | **0.48** | 0.0013 |  | 0.43 | 0.2955 |  | 0.85 | 1.0000 |
| SP_2029 | preprotein translocase, YajC subunit | 0.51 | 0.0833 |  | **0.44** | <0.0001 |  | 0.43 | 0.0510 |  | 0.65 | 0.7550 |
| SP_2030 | transketolase | 0.72 | 1.0000 |  | **0.49** | 0.0002 |  | 0.27 | 1.0000 |  | **0.46** | 0.0073 |
| SP_2040 | putative jag protein | 0.67 | 1.0000 |  | **0.52** | 0.0001 |  | 0.61 | 1.0000 |  | **0.59** | 0.0088 |
| SP_2078 | arginyl-tRNA synthetase | 0.74 | 1.0000 |  | **0.64** | 0.0023 |  | 0.58 | 1.0000 |  | 0.50 | 0.0218 |
| SP_2136 | choline binding protein PcpA | 0.38 | 1.0000 |  | 0.25 | 0.0541 |  | **0.23** | 0.0024 |  | NA | NA |
| SP_2214 | Elongation factor TS | **0.44** | <0.0001 |  | 0.51 | 0.1711 |  | 0.39 | 1.0000 |  | 0.53 | 1.0000 |
| SP_2215 | 30S Ribosomal protein S2 | **0.38** | 0.0009 |  | **0.47** | 0.0001 |  | 0.50 | 1.0000 |  | 0.39 | 0.1073 |
| SP_2223 | conserved hypothetical protein | **0.52** | 0.0006 |  | **0.54** | 0.0003 |  | 0.43 | 0.0171 |  | 0.71 | 1.0000 |
| SP_2225 | conserved hypothetical protein | 0.70 | 1.0000 |  | 0.38 | 0.3406 |  | **0.28** | 0.0084 |  | 0.63 | 1.0000 |
| * NA denotes a failure to satisfy criteria for robust microarray data. Genes meeting statistical significance are denoted in bold. | | | | | | | | | | | | |
