# Supplementary material for: Streptococcus pneumoniae in Biofilms Are Unable to Cause Invasive Disease Due to Altered Virulence Determinant Production
Source: PLoS One. 2011 Dec 8;6(12):e28738. doi: 10.1371/journal.pone.0028738 (PMC3234282; doi:10.1371/journal.pone.0028738)
Supplement: Table S2 — Genes with significantly increased expression in at least one time point during biofilm growth. (DOCX) [file pone.0028738.s005.docx]

| **Table S2.** Genes with significantly increased expression in at least one time point during biofilm growth* | | | | | | | | | | | | |
| --- | --- | --- | --- | --- | --- | --- | --- | --- | --- | --- | --- | --- |
| **TIGR4** | **Annotation** | **4 hour** | ***p* value** |  | **12 hour** | ***p* value** |  | **24 hour** | ***p* value** |  | **48 hour** | ***p* value** |
| SP_0107 | LysM domain protein | **4.27** | >0.0001 |  | 1.92 | 0.0404 |  | 2.05 | 1.0000 |  | 4.74 | 0.1493 |
| SP_0285 | Alcohol dehydrogenase, propanol-preferring. | 2.11 | 1.0000 |  | **2.54** | 0.0003 |  | 4.40 | 0.0375 |  | NA | NA |
| SP_0338 | putative ATP-dependent Clp protease, ATP-binding subunit | 4.54 | 1.0000 |  | **7.32** | 0.0020 |  | NA | NA |  | NA | NA |
| SP_0385 | Conserved hypothetical protein | 1.13 | 1.0000 |  | **1.56** | 0.0053 |  | 1.49 | 0.0540 |  | NA | NA |
| SP_0387 | DNA-binding response regulator | 1.35 | 1.0000 |  | **2.32** | 0.0028 |  | 1.57 | 1.0000 |  | NA | NA |
| SP_0392 | Degenerate transposase: SP_0392, SP_0814, SP_0850, SP_1485, SP_1593, SP_1613 | 1.77 | 1.0000 |  | **2.36** | 0.0058 |  | 4.12 | 0.0559 |  | NA | NA |
| SP_0406 | DNA mismatch repair protein | **1.50** | 0.0088 |  | 1.48 | 1.0000 |  | 1.16 | 1.0000 |  | 1.72 | 1.0000 |
| SP_0450 | Threonine desaminase | NA | NA |  | **2.13** | 0.0044 |  | NA | NA |  | NA | NA |
| SP_0516 | Heat-shock protein (activation of DnaK) | 1.67 | 0.3076 |  | 0.97 | 1.0000 |  | 1.11 | 1.0000 |  | **4.62** | 0.0005 |
| SP_0517 | Class I heat-shock protein (molecular chaperone) | **2.13** | 0.0006 |  | 1.11 | 1.0000 |  | 1.21 | 1.0000 |  | **5.33** | 0.0086 |
| SP_0646 | conserved hypothetical protein; SP_1197 | 1.92 | 0.3088 |  | **3.44** | 0.0062 |  | 5.65 | 0.0357 |  | NA | NA |
| SP_0698 | Hypothetical protein | **1.50** | 0.0100 |  | 1.24 | 1.0000 |  | 1.04 | 1.0000 |  | NA | NA |
| SP_0703 | Hypothetical protein | 1.33 | 1.0000 |  | **1.89** | 0.0002 |  | 2.89 | 0.0162 |  | 4.95 | 0.6425 |
| SP_0712 | lactate oxidase, truncation | 2.37 | 0.0369 |  | **3.05** | 0.0021 |  | 2.98 | 0.8837 |  | NA | NA |
| SP_0715 | Lactate oxidase | **2.69** | 0.0017 |  | **2.57** | 0.0011 |  | 3.10 | 0.6058 |  | 3.12 | 0.7161 |
| SP_0766 | superoxide dismutase, manganese-dependent | **3.75** | >0.0001 |  | **2.69** | 0.0004 |  | 3.42 | 1.0000 |  | NA | NA |
| SP_0799 | Sensor protein CiaH histide kinase | **3.57** | 0.0085 |  | 1.97 | 0.0339 |  | 1.36 | 1.0000 |  | 2.32 | 1.0000 |
| SP_0820 | ATP-dependent Clp protease, ATP-binding subunit ClpE | **2.71** | 0.0094 |  | 1.57 | 1.0000 |  | **2.10** | 0.0045 |  | NA | NA |
| SP_0830 | conserved hypothetical protein | 2.00 | 1.0000 |  | **1.41** | 0.0011 |  | 1.92 | 1.0000 |  | NA | NA |
| SP_0832 | hypothetical protein | 1.46 | 1.0000 |  | 1.33 | 1.0000 |  | **1.89** | 0.0050 |  | 1.69 | 1.0000 |
| SP_0842 | Pyrimidine nucleoside phosphorylase | 1.31 | 1.0000 |  | **1.69** | 0.0017 |  | 1.82 | 1.0000 |  | NA | NA |
| SP_1000 | thioredoxin family protein | **2.04** | 0.0004 |  | 1.45 | 1.0000 |  | 1.32 | 1.0000 |  | 1.83 | 1.0000 |
| SP_1110 | macrolide-efflux protein | 1.24 | 1.0000 |  | 0.95 | 1.0000 |  | 1.50 | 1.0000 |  | **2.44** | 0.0004 |
| SP_1121 | 1,4-alpha-glucan branching enzyme | NA | NA |  | **4.63** | 0.0007 |  | 5.84 | 0.1061 |  | NA | NA |
| SP_1122 | Glucose-1-phosphate adenylyltransferase | NA | NA |  | **2.39** | 0.0074 |  | 3.68 | 0.0772 |  | NA | NA |
| SP_1123 | glucose-1-phosphate adenylyltransferase, GlgD subunit | 1.57 | 1.0000 |  | **2.35** | 0.0099 |  | 2.65 | 1.0000 |  | NA | NA |
| SP_1144 | conserved hypothetical protein | NA | NA |  | 1.48 | 1.0000 |  | **2.30** | 0.0010 |  | 4.48 | 0.4010 |
| SP_1190 | Tagatose 1,6-diphosphate aldolase | 1.67 | 1.0000 |  | 1.12 | 1.0000 |  | **1.84** | 0.0001 |  | NA | NA |
| SP_1267 | Homologous to LicC, which regulates expression of LPS epitopes in H. influenzae | **1.83** | >0.0001 |  | 1.70 | 1.0000 |  | 1.40 | 1.0000 |  | **2.77** | 0.0037 |
| SP_1339 | hypothetical protein; SP_1345 | 2.09 | 1.0000 |  | 2.01 | 0.0348 |  | **6.33** | 0.0005 |  | 9.63 | 1.0000 |
| SP_1546 | Hypothetical protein | **2.28** | >0.0001 |  | 1.55 | 0.1887 |  | 1.12 | 1.0000 |  | 0.62 | 1.0000 |
| SP_1588 | oxidoreductase, pyridine nucleotide-disulfide, class I | **2.78** | 0.0022 |  | **1.70** | 0.0003 |  | 1.79 | 1.0000 |  | 1.25 | 1.0000 |
| SP_1652 | ABC transporter, permease protein | 1.20 | 1.0000 |  | **1.98** | 0.0050 |  | 2.51 | 1.0000 |  | NA | NA |
| SP_1683 | sugar ABC transporter, sugar-binding protein | 1.94 | 1.0000 |  | **2.76** | 0.0068 |  | **6.00** | 0.0086 |  | NA | NA |
| SP_1720 | Hypothetical protein | 1.38 | 1.0000 |  | 0.96 | 1.0000 |  | **1.64** | 0.0052 |  | NA | NA |
| SP_1721 | Fructokinase | 1.39 | 1.0000 |  | **2.46** | 0.0031 |  | 2.89 | 0.3096 |  | NA | NA |
| SP_1734 | rRNA methyltransferase RsmB | 0.83 | 1.0000 |  | 1.30 | 1.0000 |  | 1.14 | 1.0000 |  | **1.72** | 0.0024 |
| SP_1756 | accessory secretory protein Asp4 | NA | NA |  | **3.89** | 0.0001 |  | NA | NA |  | NA | NA |
| SP_1757 | conserved hypothetical protein GtfB | 2.19 | 1.0000 |  | **4.89** | 0.0001 |  | 6.67 | 0.0431 |  | NA | NA |
| SP_1758 | glycosyl transferase, group 1 GtfA | 2.98 | 1.0000 |  | 3.58 | 0.0541 |  | **4.89** | 0.0091 |  | NA | NA |
| SP_1760 | accessory secretory protein Asp3 | 2.52 | 1.0000 |  | **4.16** | >0.0001 |  | 6.47 | 0.0139 |  | NA | NA |
| SP_1761 | accessory secretory protein Asp2 | NA | NA |  | **5.04** | 0.0001 |  | **7.18** | 0.0047 |  | NA | NA |
| SP_1762 | accessory secretory protein Asp1 | 2.98 | 0.0358 |  | **5.09** | 0.0011 |  | 6.02 | 0.0633 |  | NA | NA |
| SP_1764 | glycosyl transferase, family 2 GlyG | 3.48 | 1.0000 |  | **4.29** | 0.0041 |  | **6.97** | 0.0005 |  | NA | NA |
| SP_1765 | glycosyl transferase, family 8 GlyF | 2.57 | 1.0000 |  | **3.51** | 0.0038 |  | **6.12** | 0.0063 |  | 8.77 | 0.0588 |
| SP_1767 | glycosyl transferase, family 8 GlyD | NA | NA |  | **4.87** | 0.0062 |  | 6.57 | 0.0415 |  | NA | NA |
| SP_1768 | nucleotide sugar synthetase like protein Nss | **3.63** | 0.0097 |  | **5.50** | 0.0018 |  | 6.12 | 0.0114 |  | NA | NA |
| SP_1771 | glycosyl transferase, family 2/glycosyl transferase family 8 GlyA | NA | NA |  | **4.43** | >0.0001 |  | **6.93** | 0.0004 |  | NA | NA |
| SP_1772 | Pneumococcal serine-rich repeat protein PsrP | NA | NA |  | **3.19** | >0.0001 |  | 2.83 | 1.0000 |  | NA | NA |
| SP_1793 | hypothetical protein | 5.64 | 0.0307 |  | **3.06** | 0.0094 |  | 7.21 | 0.1753 |  | 45.2 | 0.0161 |
| SP_1855 | alcohol dehydrogenase, zinc-containing | 1.60 | 1.0000 |  | **2.60** | 0.0063 |  | 3.92 | 1.0000 |  | NA | NA |
| SP_1906 | chaperonin GroL | **1.74** | 0.0033 |  | 0.93 | 1.0000 |  | 1.17 | 1.0000 |  | **3.46** | 0.0035 |
| SP_1948 | Hypothetical protein | NA | NA |  | 1.40 | 1.0000 |  | **2.82** | 0.0048 |  | NA | NA |
| SP_1990 | primase homolog | 1.26 | 1.0000 |  | 1.75 | 0.3790 |  | **1.66** | 0.0079 |  | NA | NA |
| SP_1996 | universal stress protein family | 2.22 | 1.0000 |  | **2.13** | 0.0015 |  | 4.53 | 0.4349 |  | NA | NA |
| SP_2026 | Alcohol-acetaldehyde dehydrogenase | 1.61 | 1.0000 |  | **2.03** | 0.0034 |  | 3.28 | 0.0304 |  | **3.05** | 0.0096 |
| SP_2063 | LysM domain protein | NA | NA |  | **4.10** | 0.0002 |  | 5.02 | 1.0000 |  | NA | NA |
| SP_2084 | ABC transporter substrate-binding protein - phosphate transport | 3.69 | 1.0000 |  | **6.64** | >0.0001 |  | NA | NA |  | NA | NA |
| SP_2087 | ABC transporter ATP-binding protein - phosphate transport | NA | NA |  | **7.01** | 0.0063 |  | 5.80 | 0.6397 |  | NA | NA |
| SP_2088 | Negative regulator of pho regulon for phosphate transport | 5.23 | 0.5209 |  | **8.22** | 0.0000 |  | 9.35 | 0.1886 |  | NA | NA |
| SP_2106 | Maltodextrin phosphorylase | **2.16** | >0.0001 |  | 0.85 | 1.0000 |  | 0.89 | 1.0000 |  | 0.81 | 1.0000 |
| SP_2123 | Positive transcriptional regulator of glucosyltransferase and Spp phenotype | 1.10 | 1.0000 |  | 0.99 | 1.0000 |  | 1.10 | 1.0000 |  | **1.87** | 0.0036 |
| SP_2148 | arginine deiminase | NA | NA |  | **4.47** | >0.0001 |  | NA | NA |  | NA | NA |
| SP_2240 | spspoJ protein | **3.59** | 0.0053 |  | 1.19 | 1.0000 |  | 1.24 | 1.0000 |  | 1.77 | 1.0000 |
| * NA denotes a failure to satisfy criteria for robust microarray data. Genes meeting statistical significance are denoted in bold. | | | | | | | | | | | | |
