# Supplementary material for: Streptococcus pneumoniae in Biofilms Are Unable to Cause Invasive Disease Due to Altered Virulence Determinant Production
Source: PLoS One. 2011 Dec 8;6(12):e28738. doi: 10.1371/journal.pone.0028738 (PMC3234282; doi:10.1371/journal.pone.0028738)
Supplement: Table S3 — Relative expression of S. pneumoniae genes following biofilm growth versus planktonic cultures as determined by microarray and qRT-PCR ΔCT values. (DOCX) [file pone.0028738.s006.docx]

| **Table S3.** Relative expression of *S. pneumoniae* genes following biofilm growth versus planktonic cultures as determined by microarray and qRT-PCR ΔCT values* | | | | | | | | | | | | | | | | | | | | |
| --- | --- | --- | --- | --- | --- | --- | --- | --- | --- | --- | --- | --- | --- | --- | --- | --- | --- | --- | --- | --- |
|  |  | **4 hour biofilms** | | | |  | **12 hour biofilms** | | | |  | **24 hour biofilms** | | | |  | **48 hour biofilms** | | | |
| **Locus** | **Gene** | Array | ±SEM | qRT-PCR | ±SEM |  | Array | ±SEM | qRT-PCR | ±SEM |  | Array | ±SEM | qRT-PCR | ±SEM |  | Array | ±SEM | qRT-PCR | ±SEM |
| SP_0117 | *pspA* | 0.60 | 0.19 | 1.01 | 0.21 |  | 0.64 | 0.16 | 1.20 | 0.17 |  | 0.56 | 0.26 | 1.16 | 0.29 |  | 1.20 | 0.42 | 1.62 | 0.50 |
| SP_0235 | *rpsK* | 1.19 | 0.21 | 0.94 | 0.20 |  | 0.72 | 0.21 | 1.15 | 0.21 |  | NA | | 0.93 | 0.31 |  | 0.08 | 0.03 | 0.44 | 0.16 |
| SP_0338 |  | 4.54 | 0.80 | 31.93 | 1.58 |  | 7.32 | 0.65 | 24.15 | 1.44 |  | NA | | 10.67 | 66.8 |  | NA | | 109.1 | - |
| SP_0419 | *fabK* | 0.25 | 0.09 | 0.20 | 0.08 |  | 0.41 | 0.12 | 0.36 | 0.14 |  | 0.44 | 0.26 | 0.29 | 0.14 |  | 0.27 | - | not done | |
| SP_0461 | *rlrA* | 0.21 | - | 0.22 | - |  | 0.31 | - | 0.23 | - |  | 0.21 | 0.13 | 0.21 | 0.06 |  | 0.22 | 0.04 | 0.20 | 0.02 |
| SP_0462 | *rgrA* | 0.37 | - | 0.39 | - |  | 0.30 | 0.07 | 0.15 | 0.05 |  | 0.45 | 0.07 | 0.20 | 0.09 |  | NA | | not done | |
| SP_0496 |  | 0.96 | 0.10 | 1.06 | 0.10 |  | 1.06 | 0.06 | 1.13 | 0.12 |  | 1.09 | 0.22 | 1.70 | 0.52 |  | NA | | not done | |
| SP_0501 |  | 0.15 | 0.08 | 0.23 | 0.08 |  | 0.19 | 0.09 | 0.35 | 0.10 |  | 0.20 | 0.10 | 0.17 | 0.18 |  | 0.35 | 0.15 | 0.54 | 0.13 |
| SP_0730 | *spxB* | 1.01 | 0.12 | 2.01 | 0.35 |  | 0.81 | 0.15 | 3.38 | 0.54 |  | 0.77 | 0.46 | 3.18 | 0.43 |  | 0.46 | 0.18 | 2.26 | 0.69 |
| SP_0943 | *gid* | 1.24 | 0.15 | 1.29 | 0.19 |  | 1.09 | 0.15 | 1.20 | 0.15 |  | 1.00 | 0.22 | 0.76 | 0.28 |  | 1.09 | 0.20 | 0.99 | 0.36 |
| SP_1269 | *licB* | 1.58 | 0.20 | 3.13 | 0.27 |  | 2.83 | 0.53 | 5.21 | 0.46 |  | 1.58 | 0.61 | 3.60 | 0.82 |  | 2.07 | 0.55 | 9.76 | 0.59 |
| SP_1427 |  | 1.14 | 0.19 | 0.78 | 0.17 |  | 1.21 | 0.10 | 0.76 | 0.15 |  | 1.26 | 0.28 | 0.92 | 0.25 |  | NA | | not done | |
| SP_1693 | *nanA* | 2.39 | 0.18 | 5.15 | 0.51 |  | 2.55 | - | 2.03 | - |  | 6.34 | - | 25.83 | - |  | NA | | not done | |
| SP_1761 | *asp2* | NA | | 8.14 | - |  | 5.04 | 0.49 | 7.75 | 0.26 |  | 7.18 | 0.96 | 16.97 | 1.07 |  | NA | | not done | |
| SP_1768 | *nss* | 3.63 | 0.26 | 6.40 | 0.28 |  | 5.50 | 0.53 | 6.21 | 0.29 |  | 6.12 | 1.10 | 13.38 | 0.45 |  | NA | | not done | |
| SP_1772 | *psrP* | NA | | 5.99 | - |  | 3.19 | 0.29 | 10.18 | 0.30 |  | 2.83 | 0.76 | 8.48 | 0.82 |  | NA | | not done | |
| SP_1793 |  | 5.64 | 0.88 | 24.55 | 1.42 |  | 3.06 | 0.41 | 15.83 | 0.88 |  | 7.21 | 1.78 | 41.54 | 2.22 |  | 45.16 | 2.93 | 224.9 | 7.76 |
| SP_1923 | *ply* | 0.35 | 0.13 | 0.50 | 0.12 |  | 0.26 | 0.09 | 0.46 | 0.16 |  | 0.39 | 0.05 | 0.70 | 0.23 |  | 0.79 | 0.00 | 0.40 | 0.16 |
| SP_2190 | *cbpA* | 1.94 | 0.33 | 2.87 | 0.46 |  | 1.16 | 0.27 | 4.72 | 0.58 |  | 1.48 | 0.28 | 10.41 | 1.43 |  | 1.51 | 0.58 | 1.23 | 0.56 |
| * NA indicates failures to satisfy criteria for robust microarray data. Dash indicates only 1 value was obtained and thus it was not possible to calculate standard error. Due to low amounts of RNA available for 48 hour samples if microarrays were inconclusive qRT-PCR was not done. | | | | | | | | | | | | | | | | | | | | |
